# Bell Inequalities with One Bit of Communication

**DOI:** 10.3390/e21020171

**Published:** 2019-02-13

**Authors:** Emmanuel Zambrini Cruzeiro, Nicolas Gisin

**Affiliations:** Department of Applied Physics, University of Geneva, 1211 Geneva, Switzerland

**Keywords:** quantum nonlocality, communication complexity

## Abstract

We study Bell scenarios with binary outcomes supplemented by one bit of classical communication. We developed a method to find facet inequalities for such scenarios even when direct facet enumeration is not possible, or at least difficult. Using this method, we partially solved the scenario where Alice and Bob choose between three inputs, finding a total of 668 inequivalent facet inequalities (with respect to relabelings of inputs and outputs). We also show that some of these inequalities are constructed from facet inequalities found in scenarios without communication, that is, the well-known Bell inequalities.

## 1. Introduction

Bell nonlocality [1,2] is one of the most intriguing phenomena encountered in modern physics. Nonlocality was discovered more than 50 years ago, and there are still simple well-posed fundamental questions about nonlocality that remain unanswered. In this article, we focus on one of these questions, which is impressively simple to state but has proven very hard to answer. In the interest of quantifying and understanding nonlocality, one can create variations of Bell’s original local hidden variable (LHV) model by adding a nonlocal resource. A nonlocal resource is any resource that establishes correlations at a distance. A PR box [3,4,5] is an example of such a nonlocal resource. Another example is classical communication [6,7,8,9,10], which is the focus of this paper. In particular, one can ask how many bits of information are needed to reproduce correlations arising from projective measurements on any two-qubit state [6,8,9,11]. For the singlet, it is known that one bit is sufficient (the explicit model is given in Reference [10]); therefore, we are interested in partially entangled states, which are known to be simulable with two bits [10], but not with zero bits [12]. We ask whether one bit also suffices to simulate projective measurements on all two-qubit partially entangled states. It is interesting that such a well-posed binary-answer question for projective measurements on two-qubit pure states has still not been answered, even though several authors have worked on this problem [13,14]. This illustrates the technical difficulty of studying nonlocality. Our strategy is to find Bell-like inequalities that are satisfied by all LHV models supplemented by one bit of communication, and then look for a violation of such inequalities. Although we do not provide an answer to Toner and Bacon’s question here, our results already provide a deeper understanding of Bell-like inequalities for scenarios with one bit of communication. Additionally, our work can be of interest to physicists working on alternative causal structures to Bell’s theorem (see References [15,16,17]).

Regular Bell scenarios and Bell scenarios supplemented with one bit of communication sent by Alice to Bob are formally described in Section 2, along with the methods we used to find the main results. In particular, we introduce a useful notation and propose a method to tackle scenarios where direct facet enumeration is difficult. Section 3 gives a proof that all projective measurements on quantum states can be reproduced by one bit of communication, for scenarios where Bob has only two dichotomic measurement settings, despite the fact that we assume the bit to be communicated from Alice to Bob. In Section 4, we discuss the results we obtained for the scenario where both Alice and Bob have three inputs. Finally, we conclude by discussing the general structure of Bell-like inequalities with one bit of communication, and future directions of research.

## 2. Bell Inequalities with Auxiliary Communication

### 2.1. Bell Scenarios

**Figure 1:**
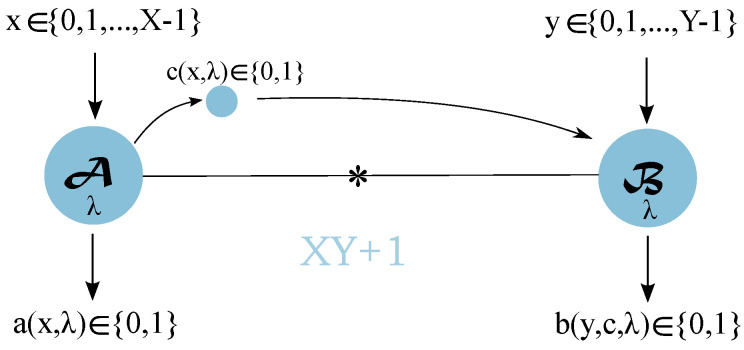
XY+1 scenario where Alice and Bob choose between X and Y binary-outcome measurements, respectively, and share local hidden variables λ (shared randomness). Alice is allowed to send one bit c(x,λ) of classical communication to Bob.

In a bipartite Bell scenario, see Figure 1, the two observers are usually called Alice and Bob. Alice and Bob choose from a set of inputs (measurement settings) and, as a result, get an output (measurement outcome). After they select their inputs, Alice and Bob are not allowed to communicate. Nevertheless, they both have access to the same set of local variables because they share randomness that was generated by a common source at a past time. The observers are allowed to use local variables to produce their outcomes. Alice and Bob both have a number of measurements settings *X* and *Y*, respectively, and a number of outputs A,B. This defines the physical setup, or Bell scenario, generally noted XYAB. Since in this article we restrict to binary-outcome measurements, we note Bell scenarios XY22 simply as XY. In the lab, Alice and Bob repetitively perform measurements and record the outcome statistics, which are described by joint probability distribution p(ab|xy). If the correlations allowed by p(ab|xy) are explainable using only common past history and local operations by the observers, physicists say the experiment statistics admit a local hidden variable (LHV model). In such a case, we can write

(1) $$p\left(ab|xy\right)=\int q\left(\lambda \right)p^{A}\left(a|x\lambda \right)p^{B}\left(b|y\lambda \right)$$

where λ is a local variable (infinite shared randomness), q(λ) is its probability distribution, and $p^{A}\left(a|x\lambda \right),p^{B}\left(b|y\lambda \right)$ are, respectively, Alice and Bob’s marginal probabilities. If Equation (1) is not satisfied, p(ab|xy) is not local.

If locality is assumed, then deterministic strategies can be defined through the marginals of Alice and Bob [2]. The marginals define their respective local strategies. Set L of all local strategies $p^{L}\left(ab|xy\right)$ is finite because Alice and Bob choose from a finite set of measurements, and it defines a convex polytope usually called the local polytope. For binary outcomes, there are $2^{X+Y}$ deterministic strategies, and the local polytope is of dimension X+Y+XY. The facets of this polytope define inequalities that are satisfied by any probability distribution in L, but are violated for quantum-probability distributions. These are the famous Bell inequalities, the simplest of which is the CHSH inequality, for binary inputs and outputs on both sides:

(2) $$\begin{array}{ll} p(00|00)+p(00|01)+ & p(00|10)-p(00|11)\\ & -p^{A}\left(0|0\right)-p^{B}\left(0|0\right)\leq 0 \end{array}$$

This inequality is violated by quantum mechanical probability distributions, up to $\frac{1}{\sqrt{2}}-\frac{1}{2}\approx 0.2071$.

### 2.2. Bell Scenarios Supplemented by One Cbit (Bell + 1)

We are interested in the simulation of projective measurements on qubits through one bit of classical communication. Since quantum correlations are symmetric with respect to Alice and Bob, we specifically consider one-way communication (in one direction; in this case, from Alice to Bob), as two-way communication would have no advantage in a quantum scenario. The protocol goes as follows: Alice and Bob first receive their inputs, then Alice is allowed to send one bit of classical communication to Bob. In this way, Alice and Bob can simulate all p(ab|xy) that satisfy:

(3) $$p\left(ab|xy\right)=\int q\left(\lambda \right)p^{A}\left(a|x\lambda \right)p^{B}\left(b|yc\lambda \right)$$

where the marginal of Bob now also depends on the value of classical bit c=c(x,λ).

One can define all local strategies with one bit of communication analogous to the original Bell scenario. The local strategies can all be written in terms of local deterministic strategies, for which the marginal probabilities of Alice and Bob can only take values 0 and 1. There is a finite number of such strategies and, hence, a finite number of vertices that define a convex polytope. Once we have generated all the vertices, we look for the facets of this polytope: this is the so-called facet-enumeration problem. We call the set of local strategies with one bit of communication C. The inequalities defining these facets are violated only if there exists a two-qubit state and projective measurements yielding correlations that cannot be reproduced using one bit of classical communication.

### 2.3. Local Strategies for Bell + 1 and Notation

Joint probability distribution p(ab|xy) for each local strategy can be computed in the following way:

(4) $$p\left(ab|xy\right)=\sum_{\lambda }q\left(\lambda \right)p^{A}\left(a|x\lambda \right)p^{B}\left(b|cy\lambda \right)$$

where c=c(x,λ) is the communication function, and can be encoded in multiple ways. In a similar fashion to Bell scenarios, we define such a scenario as XY+1, where we again omit the number of outputs as they are always binary. For a given number of inputs on Alice’s side *X*, the number of communication functions in the case of one cbit is given by the Stirling number of the second kind, denoted S(X,2) or X2, and defined as Xk:=1k!∑j=0k(−1)k−jkjjX. The Stirling number of the second kind gives the number of distinct ways to divide a set into two nonempty subsets.

By directly generating all local strategies, we obtain X2·2X·22Y vertices. This method generates repeated vertices because it takes into account the situations where Bob does not use the communication bit. By removing repetitions, we end up with a smaller number of vertices, given by:

(5) 2X2Y+X2(22Y−2Y)

This is a sum of three terms. The first term gives the vertices for the local polytope of the Bell scenario, in which case no communication function is used. The second term accounts for three kinds of strategies: Bell local strategies like the first term, strategies where there is communication but the bit is not used by Bob, and finally strategies for which the bit is used. In order to only keep the Bell local strategies and the strategies for which the bit of communication is useful, we must remove the strategies that do not use the bit, for which the third term accounts. In the second term, the Stirling number gives the number of possible communication functions, and the bit of communication gives a factor of two multiplying Y (the bit is counted as an extra binary input on Bob’s side). An interesting consequence of this is that, for different values of (X, Y), one can have the same amount of vertices. In fact, any XX+1 scenario has the same number of vertices as an (X+1)(X−1)+1 scenario. Any X(X+1)+1 scenario also has the same number of vertices as an (X+2)(X−1)+1 scenario.

The dimension of the XY+1 polytope is X+2XY. It is easy to see why: Joint probability distribution p(ab|xy) consists of 4XY elements, but none of these elements is independent due to normalization and no-signalling constraints. Normalization removes the XY of these elements, and no signalling from Alice to Bob removes X(Y−1) elements. Therefore, X+2XY is the minimal number of variables (probability elements) needed to define the polytope. The usual notation for vertices, from Toner and Bacon [11], is given by $\left\{p\left(00|xy\right)\ldots p\left(10|xy\right)\ldots p^{A}\left(a=0|x\right)\ldots \right\}$. The three dots mean that we run through all the values of x and y, for example, {p(00|xy)…} means {p(00|00),p(00|01),p(00|10),p(00|11), etc...}. We instead chose to use notation $\left\{p\left(00|xy\right)\ldots p^{B}\left(b=0|xy\right)\ldots p^{A}\left(a=0|x\right)\ldots \right\}$ similarly to Reference [18] because it makes it easier to see what inequalities reduce to when considering probability distributions in the no-signalling (NS) subspace, such as quantum probability distribution (see Table S1, provided as a Supplementary File). This becomes clear when we study the first nontrivial scenarios, 32+1 and 33+1, while 2Y+1 is trivial for all Y because Alice can simply send her input as the communication bit; in fact, as we show in Section 3, X2+1 is also trivial for all X.

A Bell + 1 inequality can be written as:

(6) $$\sum_{xy}d_{xy}p\left(00|xy\right)+\sum_{xy}e_{xy}p^{B}\left(0|xy\right)+\sum_{x}f_{x}p^{A}\left(0|x\right)\leq b$$

We can represent such an inequality as a table (see Table 1) in which elements are the coefficients multiplying each probability element $\left\{p\left(00|xy\right)\ldots p^{B}\left(b=0|xy\right)\ldots p^{A}\left(a=0|x\right)\ldots \right\}$. We denote the coefficients for p(00|xy) elements as $d_{xy}$, while the coefficients for Bob’s marginals are $e_{xy}$, and for Alice’s marginals $f_{x}$. Finally, an inequality is also characterized by its bound *b*.

Note that a vector of the form

(7) $${\vec{I}}^{S}=\left(d_{00},d_{01},\ldots ,d_{\mathrm{XY}},e_{00},e_{01},\ldots ,e_{\mathrm{XY}},c_{0},c_{1},\ldots ,c_{X}\right)$$

belongs to the NS subspace iff $e_{xy}$ is independent of *x* for all *y*.

Knowing the vertices, it is possible to compute all facets of a given polytope using dedicated software such as PORTA [19] or PANDA [20].

### 2.4. Extension of Inequalities from Bell to Bell + 1 Scenarios and Intersection of Bell + 1 Inequalities with NS Subspace

An inequality of a Bell scenario can be extended to the corresponding Bell + 1 scenario. We extend inequalities from the NS space to the one-bit space by choosing the coefficients for Bob’s marginals in a clever way. For any Bell inequality, there are infinite such extensions. We chose the one orthogonal to the NS subspace as depicted in Figure 3, i.e., we imposed that the vector characterizing the extension lay within NS subspace. This orthogonal extension is unique. Let us look at the example of 33+1, a scenario where we need to use this technique because a full resolution of the polytope is difficult. In Table 2, we show how to extend an arbitrary 33 inequality to the 33+1 space. We extended the inequality to the 33+1 space by adding coefficients for Bob’s marginals, which in this higher-dimensional space dependent on both *x* and *y*. We chose the coefficients for Bob’s marginals such that $e_{y}^{\prime }$ satisfied $e_{0y}=e_{1y}=e_{2y}=e_{y}^{\prime }/3$ for all *y*, where $e_{y}^{\prime }$ are coefficients of the 33 inequality for Bob’s marginals $p^{B}\left(0|y\right)$. In this way, one can intersect the one-bit inequality with the nonsignalling subspace and map it back to the original Bell inequality that was used for the extension.

Intersecting a one-bit inequality with NS subspace is also straightforward to do using our choice of notation, as one simply has to sum up the coefficients for Bob’s marginals $\sum_{x}e_{xy}=e_{y}^{\prime }$, then

**Figure 2:**
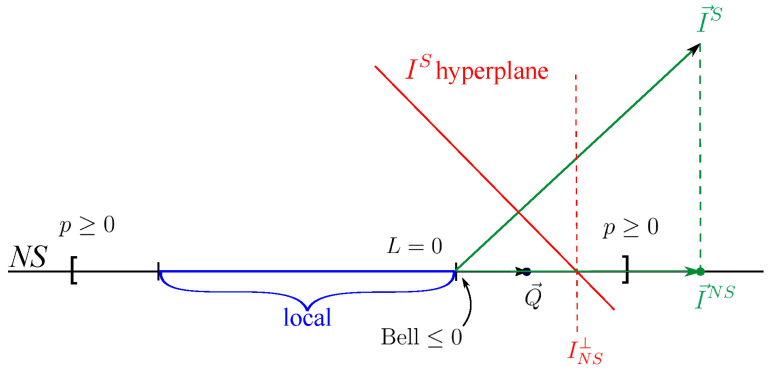
Geometry schematic of one-bit and no-signalling spaces. NS space is represented as a line, while the signalling space is represented as two-dimensional. The non-negativity conditions delimiting the NS polytope are represented by brackets.

The bound for the NS inequality in Table 3 has to be carefully considered. Indeed, this bound is the one-bit bound for ${\vec{I}}^{S}$, a particular extension (not the orthogonal one) of ${\vec{I}}^{NS}$ of Table 3. Different extensions do not give the same one-bit bound though, see Figure 2. For clarity, we used a simplified scheme. In Figure 2, we represent the signalling space as a plane containing the NS space, represented as a line. Using brackets, we also represent the bounds of the NS polytope that are given by non-negativity condition p(ab|xy)≥0 for all *a*, *b*, *x*, *y*. In a similar way, the vertical lines in the NS space delimit the local polytope. The points where those lines are placed represent facets of the local polytope. A facet of the one-bit polytope is a hyperplane $I^{S}$; in our representation, it is an interval. In order for probability distribution to not be reproducible by one bit of communication, we need its representative point to be farther to the right than the intersection of $I^{S}$ with the NS space. For any point in the NS space $\vec{q}\in$ NS, ${\vec{I}}^{S}\cdot \vec{q}={\vec{I}}^{NS}\cdot \vec{q}$. Therefore, a quantum bound for ${\vec{I}}^{NS}$ larger than the one-bit bound of ${\vec{I}}^{S}$ implies that the distribution attaining the value of the quantum bound cannot be reproduced with one bit of communication. Note that the orthogonal extension’s bound is always equal ti or larger than the correct one-bit bound, since having one bit of communication implies leaving the NS subspace.

### 2.5. Cutting the Polytope

**Figure 3:**
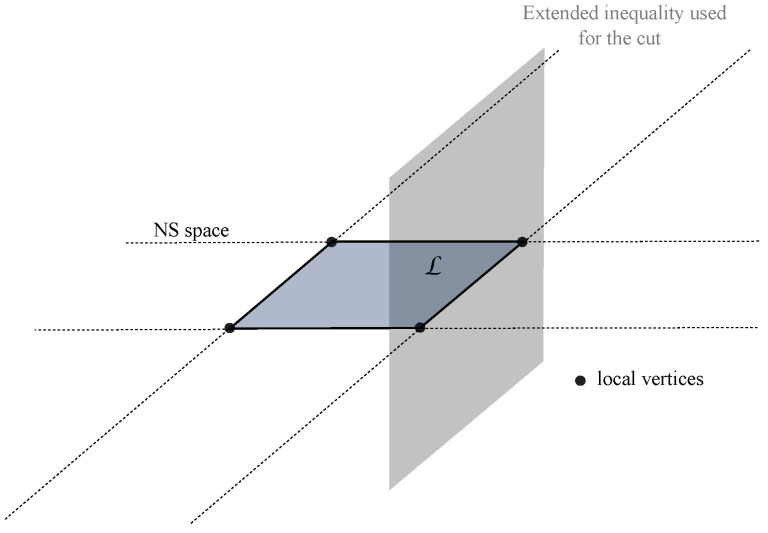
A C polytope is cut by an extended Bell inequality, which is orthogonal to the NS subspace. The NS subspace is represented as a two-dimensional space. We chose not to represent the C polytope as we did not know its geometrical form. By keeping all vertices that saturate or violate such an inequality, one obtains a subpolytope for which it is easier to find the facets via direct facet enumeration.

When direct facet enumeration cannot be done in one or two weeks, we use a trick to find a smaller set of inequalities. The trick consists in enumerating the facets for a subpolytope of C, where C is the one-bit polytope. The way we select the subpolytope is by taking a Bell scenario inequality, extending it to the one-bit space in an orthogonal way as shown in Figure 3, and removing any vertex that satisfies this new inequality. This amounts to cutting the polytope with a hyperplane.

As previously described, we chose the coefficients for Bob’s marginals in the one-bit space to be equal because this corresponds to an orthogonal extension of the facet with respect to the NS space, i.e., $I^{S}⊥\mathrm{NS}$, where $I^{S}$ is the rightmost inequality in Table 2. We extensively tested the choice of coefficients with the 32+1 scenario, which was already fully solved [14]. In order to generate all relevant facets, it is important that coefficients for Bob’s marginals for inputs that give a CHSH inequality are equal. The other coefficients seem completely arbitrary. In the 33+1 example of Table 2, for Bob’s input y=1, this means coefficients for $p^{B}\left(0|xy\right)$ for x=0,1 should be equal, and the coefficient for x=2 is arbitrary.

When we change the choice of coefficients for Bob’s marginals, we perform a rotation of the hyperplane used to cut the one-bit polytope. Therefore, one could try different choices of coefficients in order to select different sets of vertices and, therefore, produce several subpolytopes out of the original polytope. Furthermore, each relabelling of the inequality cuts a different region of the polytope, possibly revealing new facets.

There is another freedom for the cut: one can modify the bound of the inequality used for the cut. This causes a translation of the hyperplane that allows to change the size of the subpolytopes we generate. Therefore, for very hard problems, we can increase the bound to try to solve smaller subpolytopes. This translation technique has been used before (see [21] for further details).

Last but not least, when we cut a polytope and find the facets of the subpolytope, some facets are not facets of the original polytope, but they were created by the cut. In order to keep only the relevant inequalities, we check their rank and whether the vertices of the original polytope exactly saturate the inequalities bound.

## 3. *X*2 + 1 Scenarios

Scenarios of 2Y+1 are trivial because Alice can send her input as the classical bit. Surprisingly, X2+1 inequalities also cannot be violated by any NS distribution despite the assumption that the classical bit is sent from Alice to Bob. The reason is that every NS vertex of an X2 Bell scenario can be reproduced using a PR box [4,22]. Therefore, one PR box can simulate any quantum state in X2 scenarios, as boxes can be written as convex combinations of the NS vertices. Furthermore, one bit of communication is a strictly stronger nonlocal resource that one PR box [23]. Therefore, one bit of communication can simulate any quantum state in an X2 scenario.

## 4. 33 + 1 Scenario

In this section, we present our results for the 33+1 scenario. For this scenario, facet enumeration is demanding but, by cutting the polytope, we can recover a large list of inequalities. In the corresponding 33 Bell scenario, besides CHSH there is one new inequality, called $I_{3322}$, which we can also use to cut the 33+1 polytope:

$$I_{3322}=\begin{array}{cccc} & -1 & 0 & 0\\ -2 & 1 & 1 & 1\\ -1 & 1 & 1 & -1\\ 0 & 1 & -1 & 0 \end{array}\leq 0$$

### 4.1. Cutting with CHSH

We apply the cut with extended CHSH inequality using the procedure described above.

We then solve the subpolytope. We find 657 inequivalent inequalities, where 179 inequalities have a quantum advantage when intersected with the NS subspace. Note that quantum probabilities do not violate the one-bit bound *C*, but they can offer, as is the case for the 179 inequalities, an advantage with respect to local bound *L* in the NS subspace. We can distinguish the inequalities by how close the quantum bound is from the one-bit bound with the following figure of merit:

(8) $$\frac{Q-L}{C-L}$$

This figure of merit also gives a lower bound on the amount of average communication required to reproduce 3322 correlations [24]. The best quantum bound that we obtained with respect to the local bound was halfway between the local and one-bit bounds (see inequalities 195 and 232 in Table S1). This result implies that, to reproduce 3322 correlations, Alice needs to send to Bob one bit at least half of the time on average. By looking inside the NS subspace, we can show that our halfway quantum bound is obtained through a sum of two $I_{3322}$ inequalities (recall that the quantum bound of $I_{3322}$ is equal to 0.25). We also found inequalities that, in the NS subspace, correspond to the sum of two CHSH, and inequalities corresponding to one CHSH or one $I_{3322}$. In addition, we found violations that correspond to a CHSH or an $I_{3322}$ inequality, plus a term that changes the optimal state/measurement and, therefore, modifies the quantum bound, too. Performing the same analysis in the 32+1 scenario, one finds that correlations can be reproduced only if the amount of average communication is higher than 0.4142.

In Table 4, we give an explicit example of a facet that has a larger quantum bound with respect to the local bound (inequality number 232 in Table S1, which can be found in the Appendix). In the nonsignalling subspace, this facet corresponds to a sum of $I_{3322}$. In order to clarify this, we intersected the facet of the C polytope with the NS subspace.

The resulting inequality is $I_{3322}+I_{3322}^{\mathrm{perm}}$ with a bound of one instead of zero, where $I_{3322}^{\mathrm{perm}}$ is $I_{3322}$ with a relabeling of the parties (permutation of Alice and Bob labels). We found another inequality of the same type, which also includes a sum of $I_{3322}$ and $I_{3322}^{\mathrm{perm}}$, although it is less obvious to see because it also includes some other terms that do not contribute to the quantum bound. The second inequality (number 195 in Table S1) is given in Table 5.

We give more examples of 33+1 inequalities in the Appendix, along with their NS intersections.

We also tested the subpolytope method in the 32+1 scenario. By cutting with CHSH, we retrieved 80 inequalities. By removing those that are not true facets of the one-bit polytope, we obtained 17 inequalities. By sorting these inequalities into inequivalence classes, we ended up with nine inequalities, a positivity facet, and the eight new facets that were published in Reference [14]. In this scenario, by cutting the polytope we easily recover the complete list of facet inequalities. Additionally, by intersecting these facets with the NS subspace, we again find that inequalities that have a larger quantum bound than local bound are constructed from CHSH. The best inequality in terms of distance between local and quantum bounds in 32+1 is a sum of two CHSH.

### 4.2. Cutting with I3322

We repeated the “cutting” procedure using the $I_{3322}$ inequality instead of CHSH. There are two other versions (in fact many more: any relabeling as discussed in Section 2.5) of $I_{3322}$ that we can use. One of them is $I_{3322}^{\mathrm{perm}}$, which we previously introduced. The other is the symmetrized version of $I_{3322}$:

$$I_{3322}^{\mathrm{sym}}=\begin{array}{cccc} & -1 & -1 & 0\\ -1 & 0 & 1 & 1\\ -1 & 1 & -1 & 1\\ 0 & 1 & 1 & -1 \end{array}\leq 0$$

These inequalities are equivalent in the NS subspace, but when extended to the one-bit space they become inequivalent. Therefore, each cut gives a different number of vertices and facets. Cutting with $I_{3322}$, we obtained 513 inequivalent facets, 151 of them having a larger quantum bound than local bound in the NS subspace. The cut with $I_{3322}^{\mathrm{sym}}$ yields 642 inequivalent inequalities, 171 of them having a quantum advantage in the NS subspace. Finally, $I_{3322}^{\mathrm{perm}}$ gives 634 facets, 174 with a quantum advantage in the NS subspace.

We grouped all these inequalities together, and removed equivalent inequalities. We ended up with a total of 667 inequalities, 184 of which have a stronger quantum bound than local bound.

We found the same construction as before, and inequalities are constructed out of inequalities of the Bell polytope. For example, we found the same facet inequalities for 33+1 that reduce to the sum of two $I_{3322}$ in the NS subspace.

We also attempted to directly solve the full polytope. At the moment when we extracted the list of inequalities generated with the full polytope, the number of inequalities had not increased in the last two months. We thus conjecture that the list of 668 facet inequalities is complete.

## 5. Conclusions

We present a method and notation to find facets of Bell scenarios supplemented by one bit of classical communication. The notation we used simplifies the study of one-bit inequalities, especially with respect to their intersection with NS subspace. Even though the one-bit polytope is difficult to directly solve, we were able to find an extensive list of facets that we conjecture to be complete. In the 33+1 scenario, we found no quantum violation of the one-bit bound. Given the structure of 33+1 facets, and assuming our conjecture is correct, we proved that the obtained statistics by choosing between three projective measurements on any two-qubit quantum state can be reproduced by one bit of classical communication between parties. Our results also imply that, in this scenario, Alice must send one bit at least half of the time on average to Bob in order for the two parties to reproduce quantum correlations. These findings constitute a step further toward answering the binary-answer question raised in Section 1. Our results provide a better understanding of the general structure of Bell inequalities supplemented by one bit. Indeed, we found that, by intersecting the facets of the C polytope with the NS subspace, we derive inequalities that are constructed from Bell inequalities of the corresponding scenario without communication. This can be a starting point to guess new facets for scenarios where Bell inequalities are known.

The next scenarios to tackle are 34+1, 43+1, and 44+1. An important point is that our results show that the best inequalities we found in terms of distance between local and quantum bounds are sums of the same Bell inequality of the corresponding Bell scenario; for example, for 33+1, the best inequality is a sum of two Bell inequalities from 33. If this is a general trend for Bell scenarios supplemented by one bit of communication, in order to find a violation of the one-bit bound we require that Bell inequalities of the corresponding Bell scenario should be:

(1) maximally violated by a partially entangled state; and
(2) have a quantum bound that is more than halfway between local and one-bit bounds.

Only starting from four settings on one side and three on the other do we have partially entangled states maximally violating a facet Bell inequality [25]. In addition, in the 44+1 scenario, states that maximally violate Bell inequalities are, in most cases, very close to maximally entangled [25]. Furthermore, for polytopes of higher dimension than the 44 scenario [26], we still do not know the complete list of facets, which complicates the problem even more. All of these points are quite negative in the perspective of solving the binary-answer question; nevertheless, there are possible avenues to get closer to the solution. An idea is to generate facets from subpolytopes of such complicated scenarios, but one has to be lucky to find optimal inequalities in terms of communication. Another possibility is to guess inequalities using known Bell inequalities, at least up to four settings for each party.

## Figures and Tables

**Table 1:**
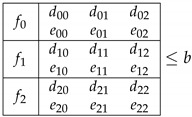
Inequalities notation 33+1. $f_{x}$ are the weights of Alice’s marginals $p_{x}^{A}\left(a=0|x\right)$, $d_{xy}$ are the weights of joint probabilities for outcomes a=b=0, and $e_{xy}$ are the coefficients for Bob’s marginals $p^{B}\left(b=0|xy\right)$.

**Table 2:**
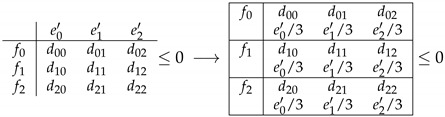
Orthogonal extension of a Bell inequality to the one-bit communication space (for example, for 33+1). The bound in both cases is the local bound.

**Table 3:**
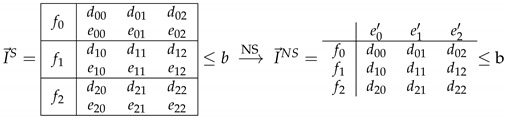
Intersecting one-bit inequality $I^{S}$ with NS subspace amounts to summing the coefficients for Bob’s marginals, characterizing one of his inputs *y*.

**Table 4:**
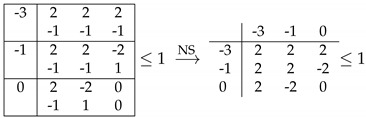
Facet of 33+1, for which the quantum bound is halfway between the local and one-bit bounds. When intersected with the NS space, this inequality reduces to a sum of $I_{3322}$ inequalities. This inequality corresponds to facet number 232 in Table S1.

**Table 5:**
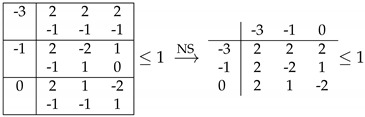
Second facet (number 195) of 33+1 for which the quantum bound is halfway between the local and one-bit bounds.

**Table A1:**
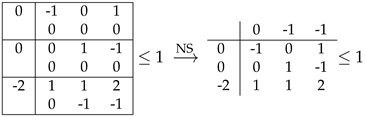
Facet of 33+1, for which the quantum bound is $\sqrt{2}-1$, for a local bound of zero and a one-bit bound of one. When intersected with the NS space, this inequality reduces to a sum of CHSH inequalities.

**Table A2:**
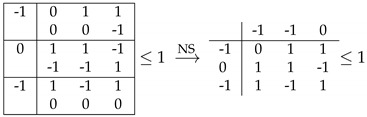
Facet of 33+1, for which the quantum bound is 0.25, for a local bound of zero and a one-bit bound of one. When intersected with the NS space, this inequality reduces to $I_{3322}$. In fact, we see that it corresponds to $I_{3322}^{\mathrm{sym}}$ if we permute Alice’s inputs x=1 and x=2. This inequality is maximally violated by the maximally entangled state, and its quantum bound is the $I_{3322}$ quantum bound.

**Table A3:**
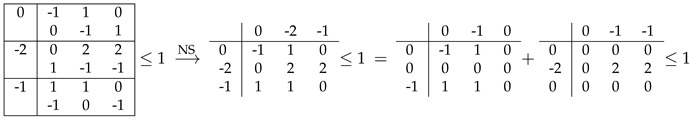
Facet of 33+1, for which the quantum bound is $1/2(\sqrt{2}-1)$, for a local bound of zero and a one-bit bound of one. When intersected with the NS space, this inequality reduces to a CHSH inequality for two of each party’s inputs and some other terms. This inequality is maximally violated by the maximally entangled state, and its quantum bound is the CHSH quantum bound.

**Table A4:**
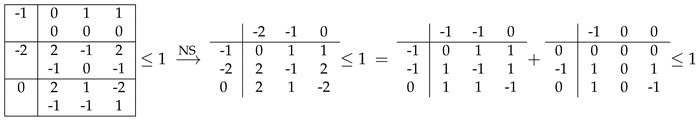
Facet of 33+1, for which the quantum bound is 0.4158, for a local bound of zero and a one-bit bound of one. When intersected with the NS space, this inequality reduces to a sum of a CHSH inequality for two of each party’s inputs and an $I_{3322}$. This inequality is maximally violated by the nonmaximally entangled state.

## Supplementary Materials

The following are available online at http://www.mdpi.com/1099-4300/21/2/171/s1. Table S1: Conjectured complete list of tight Bell + 1 inequalities with three settings for both parties. Coefficients for each inequality are given in the following order: $d_{00}$$d_{01}$$d_{02}$$d_{10}$$d_{11}$$d_{12}$$d_{20}$$d_{21}$$d_{22}$$e_{00}$$e_{01}$$e_{02}$$e_{10}$$e_{11}$$e_{12}$$e_{20}$$e_{21}$$e_{22}$$f_{0}$$f_{1}$$f_{2}$. For each inequality, we give local bound *L*, two-qubit quantum bound *Q*, one bit of communication bound *C*, and quantum state that achieves the largest quantum bound $|\psi \left(\theta_{\max}\right)\rangle =\cos\theta_{\max}|00\rangle +\sin\theta_{\max}|11\rangle$. All quantities were computed for nondegenerate measurements.

## Appendix A. Examples of 33 + 1 Facets and Complete List

In this appendix, we give examples of 33+1 facets, along with their NS intersection and connection to Bell inequalities. We start with a 33+1 facet, shown in Table A1, that reduces to the sum of two CHSH inequalities in the NS subspace (inequality 349 in our Table S1).

This inequality corresponds, in the NS subspace, to a sum of one CHSH inequality that uses Alice’s inputs x=0,2 and Bob’s inputs y=0,2, and another CHSH that uses x=1,2 and y=1,2. Therefore, the quantum bound of this inequality is $\sqrt{2}-1\approx 0.4142$, which is twice the amount of violation for CHSH. The quantum bound is obtained for the maximally entangled state $1/\sqrt{2}(|00\rangle +\left|11\rangle \right)$.

One can also have a single $I_{3322}$ contained in the facet, as the example in Table A2 shows (inequality number 529):

In Table A3, we give an example of a facet which when intersected with the NS space reduces to a CHSH inequality and some other terms. Despite the extra terms, its quantum bound is the maximum violation of CHSH, attained for a maximally entangled state. This inequality is number 380 in Table S1, and it is similar to the inequality of Table 5, in the sense that both are constructed from Bell inequalities, and have some extra terms that do not contribute to the quantum bound. If we remove these extra terms, the quantum and local bounds would therefore not change. Understanding how these extra terms arise could lead to a better understanding of how to construct Bell + 1 inequalities from Bell inequalities.

Most inequalities of 33+1 have a quantum bound that is different than the CHSH bound, $I_{3322}$ or twice their amount. Most inequalities have quantum bounds that do not easily relate to Bell inequalities for binary outcomes, up to three settings. As a final example, we show such an 33+1 facet and how its NS intersection is constructed from CHSH and $I_{3322}$ even if the quantum bound does not directly relate to the maximal violations of the Bell inequalities. One such facet is inequality number 196 in Table S1.

As shown in Table A4, facet number 196 corresponds to a sum of $I_{3322}^{\mathrm{sym}}$ and CHSH using inputs x=1,2 of Alice and y=0,2 of Bob. maximum violation of 0.4158 is given by a partially entangled state:

(A1) |ψ〉=0.738|00〉+0.675|11〉

and resistance to noise for this inequality is λ=0.7830, larger than the resistance to noise of CHSH ($\lambda_{\mathrm{CHSH}}=0.7071$), but lower than the resistance to noise of $I_{3322}$ ($\lambda_{I_{3322}}=0.8$).
