# Supplementary material for: Bell Inequalities with One Bit of Communication
Source: Entropy (Basel). 2019 Feb 13;21(2):171. doi: 10.3390/e21020171 (PMC7514653; doi:10.3390/e21020171)
Supplement: Supplementary file 1 [file entropy-21-00171-s001.zip › entropy-416857-supplymentary/tableS1.pdf]

Table S1. Conjectured complete list of tight Bell+1 inequalities with three settings for both parties. The coefficients for each inequality are given in the following order d_{00} d_{01} d_{02} d_{10} d_{11} d_{12} d_{20} d_{21} d_{22} e_{00} e_{01} e_{02} e_{10} e_{11} e_{12} e_{20} e_{21} e_{22} f_0 f_1 f_2. For each inequality we give the local bound L, the two-qubit quantum bound Q, the one bit of communication bound C and the quantum state that achieves the largest quantum bound $|\psi(\theta_{\max})\rangle = \cos\theta_{\max}|00\rangle + \sin\theta_{\max}|11\rangle$. All quantities are computed for non-degenerate measurements.

#	Ineq	L	Q	C	$\theta_{\max}/\pi$
1	-9 6 8 9 1 9 4 6 -5 3 -5 -5 -3 -1 -3 -1 -1 -1 0 -10 -4	1	1	4	0
2	-8 6 8 8 -2 8 4 6 -2 3 -5 -5 -3 -1 -3 -1 -1 -1 0 -8 -4	1	1	4	0
3	-8 6 8 8 0 8 4 6 -4 3 -5 -5 -3 -1 -3 -1 -1 -1 0 -8 -4	1	1	4	0
4	-7 5 6 7 1 6 3 5 -4 3 -4 -4 -3 -1 -2 -1 -1 0 0 -7 -3	1	1	3	0
5	-6 5 6 6 -1 6 3 5 -2 3 -4 -4 -3 -1 -2 -1 -1 0 0 -6 -3	1	1	3	0
6	-6 5 6 6 0 6 3 5 -3 3 -4 -4 -3 -1 -2 -1 -1 0 0 -6 -3	1	1	3	0
7	-5 2 5 0 0 0 5 0 5 1 -2 -2 1 2 -3 -1 -2 -3 0 0 -5	1	1	3	0
8	-5 2 5 0 1 -1 5 0 5 1 -2 -2 1 1 -2 -1 -2 -3 0 0 -5	1	1	3	0
9	-5 2 5 1 2 -2 5 0 5 1 -2 -2 0 0 -1 -1 -2 -3 0 0 -5	1	1	3	0
10	-5 2 5 5 1 6 0 0 0 1 -2 -2 -1 -2 -3 1 2 -3 0 -6 0	1	1	3	0
11	-5 2 5 5 1 6 0 1 -1 1 -2 -2 -1 -2 -3 1 1 -2 0 -6 0	1	1	3	0
12	-5 2 5 5 1 6 1 2 -2 1 -2 -2 -1 -2 -3 0 0 -1 0 -6 0	1	1	3	0
13	-5 2 5 5 2 7 1 2 -3 1 -2 -2 -1 -2 -3 0 0 -1 0 -7 0	1	1	3	0
14	-5 2 7 5 2 5 0 0 0 1 -2 -3 -1 -2 -2 -1 2 -4 -2 -5 0	0	0	2	0
15	-5 2 7 5 2 5 0 2 -2 1 -2 -3 -1 -2 -2 -1 0 -2 -2 -5 0	0	0	2	0
16	-5 3 6 5 0 5 2 2 0 1 -3 -3 -1 -1 -2 -1 1 -3 -1 -5 -2	0	0	2	0
17	-5 3 6 5 0 5 2 3 -1 1 -3 -3 -1 -1 -2 -1 0 -2 -1 -5 -2	0	0	2	0
18	-5 4 4 4 -3 4 5 5 1 1 -3 -3 -1 -1 -1 -1 -1 -1 0 -4 -6	0	0.0899	2	0.1448
19	-5 4 6 5 1 5 2 4 -3 1 -3 -3 -1 -1 -1 -1 -1 -1 -2 -6 -2	0	0	2	0
20	-5 5 7 5 0 5 3 3 0 2 -4 -4 -2 -1 -1 -2 1 -3 -2 -5 -3	0	0	2	0
21	-5 5 7 5 0 5 3 5 -2 2 -4 -4 -2 -1 -1 -2 -1 -1 -2 -5 -3	0	0.0045	2	0.2044
22	-5 6 7 5 2 5 3 6 -6 1 -2 -3 -1 -2 -2 -4 -4 2 -6 -5 0	0	0.044	2	0.1871
23	-5 7 7 4 -4 7 5 5 0 2 -4 -4 -3 1 -3 -2 -1 -1 -4 -3 -5	0	0.0844	2	0.2235
24	-4 1 5 4 2 4 -1 2 -2 1 -1 -2 -1 -2 -2 0 0 -1 -1 -4 0	0	0.3467	2	0.1895
25	-4 2 4 4 0 4 1 1 0 1 -2 -2 -1 -1 -2 0 1 -2 0 -4 -1	0	0.3106	2	0.1655
26	-4 2 4 4 0 4 1 2 -1 1 -2 -2 -1 -1 -2 0 0 -1 0 -4 -1	0	0.3033	2	0.1639
27	-4 2 6 0 0 0 0 2 -2 1 -1 -2 3 -1 2 -1 -1 -2 -2 0 0	3	3	5	0
28	-4 2 6 0 2 -2 2 0 2 1 -1 -2 -1 -1 -2 1 -1 0 -2 0 -2	1	1	3	0
29	-4 3 2 2 0 2 2 3 -1 2 -2 -1 -2 -1 -1 0 -1 1 0 -2 -2	1	1	2	0
30	-4 3 3 2 -3 3 4 3 1 2 -2 -2 -1 1 -1 -2 -1 -1 0 -1 -4	1	1.0667	2	0.2167
31	-4 3 3 2 -1 3 2 3 -1 2 -2 -1 -2 0 -2 0 -1 1 -1 -2 -2	1	1.1019	2	0.2144
32	-4 3 3 3 -1 3 3 3 -1 2 -2 -2 -2 -1 -1 -1 -1 1 0 -3 -2	1	1	2	0
33	-4 3 3 4 1 3 1 1 -1 2 -2 -2 -2 -1 -1 0 1 -1 0 -4 -1	1	1	2	0
34	-4 3 3 4 1 3 1 2 -2 2 -2 -2 -2 -1 -1 0 0 0 0 -4 -1	1	1	2	0
35	-4 3 4 -1 -1 0 2 3 -1 2 -2 -2 2 0 2 -2 -1 -1 -1 1 -2	3	3	4	0
36	-4 3 4 2 1 1 2 3 -1 2 -2 -2 0 -1 1 -2 -1 -1 -1 -2 -2	1	1	2	0
37	-4 3 4 3 -4 4 4 4 1 2 -2 -2 -2 2 -2 -2 -2 -1 -1 -1 -4	1	1	2	0
38	-4 3 4 3 -2 4 3 3 -1 2 -2 -2 -2 0 -2 -1 -1 1 -1 -3 -2	1	1.0505	2	0.2262
39	-4 3 5 2 3 3 2 5 -3 0 -1 -1 2 -3 1 -2 -2 -1 -3 -5 -2	1	1	3	0
40	-4 3 5 3 1 3 1 3 -2 1 -2 -2 0 -1 0 -1 -1 -1 -2 -4 -1	0	0.365	2	0.208
41	-4 3 6 1 3 -2 3 -1 2 1 -2 -2 -1 -1 -2 0 -1 0 -2 -1 -2	1	1	3	0
42	-4 4 0 2 0 2 0 0 0 1 -1 1 -3 -3 -1 3 1 -1 0 -2 0	2	2	4	0

43	-4 4 0 2 0 2 2 2 -2 1 -1 1 -3 -3 -1 1 -1 1 0 -2 0	2	2	4	0
44	-4 4 0 2 2 4 4 6 -4 1 -1 1 -1 -3 -3 -1 -3 3 0 -4 -2	2	2	4	0
45	-4 4 0 4 2 2 0 0 0 1 -1 1 -3 -3 -1 3 1 -1 0 -4 0	2	2	4	0
46	-4 4 0 4 2 2 2 2 -2 1 -1 1 -3 -3 -1 1 -1 1 0 -4 0	2	2	4	0
47	-4 4 0 4 2 4 4 4 -4 1 -1 1 -3 -3 -3 -1 -3 3 0 -4 0	2	2.1102	4	0.1491
48	-4 4 5 4 0 4 2 4 -2 2 -3 -3 -2 -1 -1 -1 -1 0 -1 -4 -2	0	0.4426	2	0.2234
49	-4 4 5 5 -1 5 3 4 -2 1 -3 -3 -2 0 -2 -1 -1 -1 -1 -4 -3	0	0	2	0
50	-4 4 6 4 0 4 2 2 0 1 -3 -3 -1 -1 -1 -1 1 -3 -2 -4 -2	0	0	2	0
51	-4 4 6 6 -2 6 4 4 -2 1 -3 -3 -3 1 -3 -1 -1 -1 -2 -4 -4	0	0	2	0
52	-4 4 7 4 1 4 1 4 -3 1 -3 -3 -1 -2 -1 -1 -1 -1 -3 -4 -1	0	0	2	0
53	-4 4 7 7 -3 7 5 5 -2 1 -3 -3 -4 2 -4 -1 -2 -1 -3 -4 -5	0	0	2	0
54	-4 4 8 0 0 0 2 6 -4 1 -1 -2 3 -3 2 -3 -3 -2 -4 0 -2	2	2	5	0
55	-4 4 8 2 6 -4 2 -2 2 1 -1 -2 -3 -3 -2 1 -1 0 -4 -2 0	2	2	5	0
56	-4 5 7 2 -1 3 2 5 -3 2 -3 -3 0 -1 0 -2 -2 -1 -3 -2 -2	1	1	3	0
57	-4 6 6 4 -4 6 4 4 0 1 -3 -3 -3 1 -3 -1 -1 -1 -4 -2 -4	0	0.1239	2	0.2228
58	-4 6 7 5 -5 7 5 5 0 1 -3 -3 -4 2 -4 -1 -2 -1 -5 -2 -5	0	0.0913	2	0.2154
59	-3 -2 -3 5 -2 5 5 2 -5 0 4 4 -3 -2 -1 -2 -2 1 5 -5 0	6	6	8	0
60	-3 0 3 -1 0 -1 1 0 1 1 0 -1 0 0 -1 1 0 0 0 1 -1	2	2	3	0
61	-3 0 3 0 0 0 -1 0 -1 1 0 -1 2 0 1 0 0 -1 0 0 1	3	3	4	0
62	-3 0 3 0 0 0 0 0 0 1 0 -1 2 0 1 -1 0 -2 0 0 0	2	2	3	0
63	-3 0 3 1 -1 1 0 0 0 1 -1 -2 1 1 1 -1 1 -1 0 -1 0	2	2	3	0
64	-3 0 3 2 1 2 -1 1 -1 1 0 -1 0 -1 -1 0 0 -1 0 -2 0	1	1	2	0
65	-3 0 3 3 1 3 0 0 0 1 -1 -2 -1 -1 -1 -1 1 -1 0 -3 0	0	0	1	0
66	-3 0 3 3 2 3 -1 2 -2 1 0 -1 -1 -2 -2 0 0 0 0 -3 0	1	1	2	0
67	-3 1 2 2 0 2 1 0 1 1 -1 0 -1 -1 -2 1 1 -1 0 -2 -1	1	1.0391	2	0.1059
68	-3 1 2 2 0 2 1 1 0 1 -1 0 -1 -1 -2 1 0 0 0 -2 -1	1	1.0053	2	0.0616
69	-3 1 2 3 -1 2 1 1 0 1 -1 0 -2 0 -2 1 0 0 0 -2 -1	1	1.0117	2	0.1016
70	-3 1 3 0 -1 -1 1 1 0 1 -1 -1 2 0 2 -1 0 -2 0 1 -1	3	3	4	0
71	-3 1 3 0 0 0 1 1 0 1 -1 -1 2 -1 1 -1 0 -2 0 0 -1	2	2	3	0
72	-3 1 3 0 0 0 4 0 4 0 -1 -1 1 1 -2 -1 -1 -2 0 0 -4	0	0.2003	2	0.1594
73	-3 1 3 0 1 -1 1 0 1 1 0 -1 -1 -1 -1 1 0 0 -1 0 -1	1	1.0099	2	0.0873
74	-3 1 3 0 1 -1 1 1 0 1 0 -1 -1 -1 -1 1 -1 1 -1 0 -1	1	1.0641	2	0.1558
75	-3 1 3 1 0 1 1 1 0 1 -1 -1 1 -1 0 -1 0 -2 0 -1 -1	1	1	2	0
76	-3 1 3 1 1 -1 3 0 3 1 -1 -1 0 0 0 -1 -1 -2 0 0 -3	1	1	2	0
77	-3 1 3 1 1 -1 4 0 4 0 -1 -1 0 0 -1 -1 -1 -2 0 0 -4	0	0.1772	2	0.1512
78	-3 1 3 1 1 0 2 -1 1 1 -1 -1 -1 0 -2 0 0 0 0 -1 -1	1	1	2	0
79	-3 1 3 1 1 0 4 -1 3 1 -1 -1 1 0 -1 -2 0 -2 0 -1 -3	1	1	2	0
80	-3 1 3 1 1 2 1 1 0 1 -1 -1 1 -1 0 -1 0 -2 0 -2 -1	1	1	2	0
81	-3 1 3 2 1 -1 4 -1 3 1 -1 -1 0 0 0 -2 0 -2 0 -1 -3	1	1	2	0
82	-3 1 3 3 -1 3 1 1 1 1 -1 -1 -1 -1 -2 1 1 -1 0 -3 -2	1	1	2	0
83	-3 1 4 0 0 0 1 1 0 1 0 -1 2 -1 1 -2 0 -3 -1 0 -1	1	1	3	0
84	-3 1 4 0 0 0 1 2 -1 1 0 -1 2 -1 1 -2 -1 -2 -1 0 -1	1	1.0595	3	0.1514
85	-3 1 4 0 0 0 5 0 5 -1 -1 -1 1 1 -3 -1 -1 -2 -1 0 -5	0	0	2	0
86	-3 1 4 1 1 -1 5 0 5 -1 -1 -1 0 0 -2 -1 -1 -2 -1 0 -5	0	0	2	0
87	-3 1 4 1 1 0 1 -1 1 1 0 -1 -2 0 -3 1 0 0 -1 -1 0	1	1	3	0
88	-3 1 4 1 2 -1 1 -1 1 1 0 -1 -2 -1 -2 1 0 0 -1 -1 0	1	1.084	3	0.1846
89	-3 1 4 3 1 3 -1 3 -3 2 0 -2 -2 -2 -1 -1 -1 1 -1 -3 0	1	1	2	0
90	-3 1 4 3 1 3 0 0 0 1 -1 -2 -1 -1 -1 -1 1 -2 -1 -3 0	0	0	1	0
91	-3 2 1 1 0 1 1 0 1 1 -1 0 -1 -1 -1 1 1 -1 0 -1 -1	1	1.0811	2	0.1667
92	-3 2 1 1 0 1 1 1 0 1 -1 0 -1 -1 -1 1 0 0 0 -1 -1	1	1.0099	2	0.0879

93	-3 2 1 1 0 1 2 2 -1 1 -1 0 -1 -1 -1 0 -1 1 0 -1 -1	1	1.0168	2	0.1554
94	-3 2 1 3 2 3 1 2 -3 1 -1 0 -1 -1 -1 0 -1 1 0 -5 0	1	1.0719	2	0.2269
95	-3 2 2 2 -3 2 3 3 1 1 -1 -1 -1 1 -1 -1 -1 -1 0 0 -4	1	1.0698	2	0.2233
96	-3 2 2 2 -1 2 1 1 0 1 -1 0 -2 0 -2 1 0 0 -1 -1 -1	1	1.0057	2	0.0966
97	-3 2 2 2 -1 2 1 1 1 1 -1 -1 -1 -1 -1 1 1 -1 0 -2 -2	1	1	2	0
98	-3 2 2 2 -1 2 2 2 -1 1 -1 0 -2 0 -2 0 -1 1 -1 -1 -1	1	1.0967	2	0.1829
99	-3 2 2 2 -1 2 3 3 -1 1 -1 -1 -1 -1 -1 -1 -1 1 0 -2 -2	1	1	2	0
100	-3 2 3 -1 -1 -1 1 1 0 1 -1 -1 2 0 2 -1 0 -2 -1 2 -1	3	3	4	0
101	-3 2 3 -1 -1 -1 1 2 -1 1 -1 -1 2 0 2 -1 -1 -1 -1 2 -1	3	3	4	0
102	-3 2 3 0 0 0 1 2 -2 1 0 -1 2 0 1 -2 -2 0 -2 0 0	2	2	3	0
103	-3 2 3 1 0 1 1 2 -2 1 0 -1 1 0 0 -2 -2 0 -2 -1 0	1	1.0031	2	0.0926
104	-3 2 3 3 -2 3 3 2 1 1 -2 -2 -1 0 -1 -1 0 -1 0 -3 -3	0	0.1438	1	0.1796
105	-3 2 3 3 -1 3 1 2 -1 1 -2 -2 -1 0 -1 0 0 0 0 -3 -1	1	1	2	0
106	-3 2 3 3 -1 3 2 2 0 1 -2 -2 -1 0 -1 -1 0 -1 0 -3 -2	0	0.0772	1	0.1441
107	-3 2 3 3 0 3 2 2 -1 1 -2 -2 -1 0 -1 -1 0 -1 0 -3 -2	0	0.0584	1	0.1316
108	-3 2 3 3 2 3 1 4 -4 1 0 -1 -1 -2 -2 -2 -2 2 -2 -3 0	1	1	2	0
109	-3 2 3 5 2 5 3 4 -5 -1 0 0 -1 -2 -1 -2 -2 1 -2 -7 0	1	1	3	0
110	-3 2 4 0 0 0 1 3 -2 1 0 -1 2 -1 1 -2 -2 -1 -2 0 -1	1	1.1966	3	0.1881
111	-3 2 4 1 3 -2 1 -1 1 1 0 -1 -2 -2 -1 1 0 0 -2 -1 0	1	1.253	3	0.2063
112	-3 2 4 3 1 3 1 1 0 1 -2 -2 -1 -1 -1 -1 1 -2 -1 -3 -1	0	0	1	0
113	-3 2 4 3 1 3 1 2 -1 1 -2 -2 -1 -1 -1 -1 0 -1 -1 -3 -1	0	0	1	0
114	-3 2 4 4 -1 4 3 3 -1 1 -2 -2 -2 1 -2 -1 -1 -1 -1 -3 -3	0	0	1	0
115	-3 2 5 0 0 0 5 -2 3 -1 -2 -2 1 2 -3 -1 0 -1 -2 0 -3	1	1	3	0
116	-3 2 5 0 1 -1 5 -2 3 -1 -2 -2 1 1 -2 -1 0 -1 -2 0 -3	1	1	3	0
117	-3 2 5 1 2 -2 5 -2 3 -1 -2 -2 0 0 -1 -1 0 -1 -2 0 -3	1	1	3	0
118	-3 2 5 2 4 -2 1 0 -1 1 0 -1 -3 -2 -2 2 -2 2 -2 -2 0	2	2	4	0
119	-3 3 0 2 2 3 3 3 -3 1 -1 0 -1 -2 -2 -1 -2 2 0 -3 -1	1	1	2	0
120	-3 3 0 3 3 4 3 3 -4 1 -1 0 -1 -2 -2 -1 -2 2 0 -5 -1	1	1.0504	2	0.2267
121	-3 3 1 1 -1 0 4 3 -1 1 -1 -1 1 0 1 -2 -2 0 0 0 -3	2	2	3	0
122	-3 3 1 2 1 -1 2 1 1 1 -1 -1 0 0 0 -1 -2 0 0 -1 -2	1	1	2	0
123	-3 3 1 3 2 2 2 1 -2 1 -1 -1 -1 -2 0 0 0 0 0 -4 -1	1	1	2	0
124	-3 3 1 3 2 4 2 3 -4 1 -1 0 -1 -1 -2 -1 -2 2 -1 -5 0	1	1.0667	2	0.2169
125	-3 3 2 -1 -1 0 3 3 -2 1 -2 -2 2 0 2 -1 -1 0 0 1 -3	3	3	4	0
126	-3 3 2 0 -1 1 3 3 -1 1 -2 -2 1 0 1 -1 -1 0 0 0 -3	2	2	3	0
127	-3 3 2 0 0 0 3 3 0 1 -2 -2 1 -2 2 -1 -1 0 0 0 -3	2	2	3	0
128	-3 3 2 1 -1 1 3 3 0 1 -2 -2 0 -1 1 -1 -1 0 0 -1 -3	1	1	2	0
129	-3 3 2 3 2 2 1 1 -2 1 -1 -1 -1 -2 0 0 0 0 -1 -4 0	1	1	2	0
130	-3 3 2 6 1 6 5 4 -6 -1 0 0 -2 -1 -2 -3 -3 2 -2 -7 0	1	1	3	0
131	-3 3 3 0 -1 -1 2 3 -2 1 -1 -1 2 0 2 -2 -2 0 -2 1 -1	3	3	4	0
132	-3 3 3 0 -1 1 3 3 0 2 -2 -2 1 0 1 -2 -1 -1 0 0 -3	2	2	3	0
133	-3 3 3 1 -1 2 3 3 0 2 -2 -2 0 0 0 -2 -1 -1 0 -1 -3	1	1	2	0
134	-3 3 3 1 1 2 2 3 -2 1 -1 -1 1 -1 0 -2 -2 0 -2 -2 -1	1	1	2	0
135	-3 3 3 2 -2 3 3 3 0 2 -2 -2 -1 1 -1 -2 -1 -1 0 -1 -3	1	1	2	0
136	-3 3 3 3 0 3 0 0 0 2 -2 -2 -2 -1 -1 1 2 -1 0 -3 0	2	2	3	0
137	-3 3 4 -1 -1 0 1 3 -2 1 -2 -2 2 0 2 -1 -1 0 -2 1 -1	3	3	4	0
138	-3 3 4 1 -1 2 2 3 -1 2 -2 -2 0 0 0 -2 -1 -1 -1 -1 -2	1	1	2	0
139	-3 3 4 2 2 2 1 1 0 0 -1 -1 1 -2 1 -1 1 -3 -3 -4 -1	0	0.0304	2	0.1301
140	-3 3 4 2 2 2 1 3 -2 0 -1 -1 1 -2 1 -1 -1 -1 -3 -4 -1	0	0.2518	2	0.1976
141	-3 3 4 3 -3 4 4 4 0 2 -2 -2 -2 2 -2 -2 -2 -1 -1 -1 -4	1	1	2	0
142	-3 3 4 4 -1 4 3 3 -2 1 -2 -2 -2 1 -2 -1 -1 0 -2 -3 -3	0	0	1	0

143	-3 3 5 2 0 2 1 3 -2 1 -2 -2 0 -1 0 -1 -1 -1 -2 -2 -1	0	0.2625	2	0.2072
144	-3 4 1 3 2 1 0 0 0 1 -1 0 -2 -3 0 2 1 -1 -1 -3 0	1	1.133	3	0.1829
145	-3 4 1 3 2 1 1 1 -1 1 -1 0 -2 -3 0 1 0 0 -1 -3 0	1	1.1313	3	0.1974
146	-3 4 1 4 1 4 4 4 -4 1 -1 0 -2 -2 -3 -2 -3 3 -1 -4 0	1	1.4021	3	0.1987
147	-3 4 2 4 0 4 4 4 -4 1 -1 0 -2 -1 -3 -2 -3 3 -2 -4 0	1	1.4667	3	0.2054
148	-3 4 3 2 -1 3 0 0 0 2 -2 -2 -2 -1 -1 1 2 -1 -1 -2 0	2	2	3	0
149	-3 4 3 2 -1 3 1 1 0 2 -2 -2 -2 -1 -1 0 1 -1 -1 -2 -1	1	1	2	0
150	-3 4 4 3 3 3 2 2 -1 -1 -1 -1 1 -3 1 -1 1 -3 -4 -6 -2	0	0	2	0
151	-3 4 4 3 3 3 2 4 -3 -1 -1 -1 1 -3 1 -1 -1 -1 -4 -6 -2	0	0	2	0
152	-3 5 5 3 3 3 1 1 0 -1 -2 -1 1 -3 1 -1 2 -4 -5 -6 -1	0	0	2	0
153	-3 5 5 3 3 3 1 4 -3 -1 -2 -1 1 -3 1 -1 -1 -1 -5 -6 -1	0	0	2	0
154	-2 -2 0 0 0 0 0 0 0 0 1 1 2 -1 1 0 1 -1 2 0 0	4	4	5	0
155	-2 -2 0 2 0 2 0 0 0 0 1 1 0 -1 -1 0 1 -1 2 -2 0	2	2	3	0
156	-2 -2 0 4 -1 4 0 0 0 -1 2 2 -1 -1 -2 1 1 -2 2 -4 0	2	2.0766	4	0.1627
157	-2 -2 0 4 -1 4 1 1 -1 -1 2 2 -1 -1 -2 0 0 -1 2 -4 0	2	2.173	4	0.2053
158	-2 -2 2 6 0 6 2 0 -2 -2 1 1 -2 -1 -3 0 1 -1 0 -6 0	1	1	3	0
159	-2 -1 -2 1 -1 1 3 2 -2 0 2 2 0 -1 0 -1 -1 0 3 -1 -2	3	3	4	0
160	-2 -1 -2 2 -1 2 3 1 -2 0 2 2 -1 -1 0 -1 -1 0 3 -2 -1	3	3	4	0
161	-2 -1 1 2 1 3 1 0 1 0 1 1 0 -2 -2 0 2 -2 1 -3 -1	2	2	3	0
162	-2 -1 1 4 -1 3 2 1 -1 0 1 1 -2 0 -2 0 0 0 1 -3 -1	2	2	3	0
163	-2 -1 1 4 0 4 0 0 0 -1 1 1 -1 -1 -2 1 1 -2 1 -4 0	1	1.0162	3	0.1623
164	-2 -1 1 4 0 4 1 1 -1 -1 1 1 -1 -1 -2 0 0 -1 1 -4 0	1	1.1763	3	0.2174
165	-2 0 2 0 0 0 -1 0 -1 1 0 -1 1 0 1 0 0 0 0 0 1	2	2.1547	3	0.1959
166	-2 0 2 0 0 0 4 0 4 -1 0 0 1 1 -2 -1 -1 -2 0 0 -4	0	0	2	0
167	-2 0 2 1 0 1 -1 0 -1 1 0 -1 0 0 0 0 0 0 0 -1 1	1	1.4142	2	0.25
168	-2 0 2 1 1 -1 4 0 4 -1 0 0 0 0 -1 -1 -1 -2 0 0 -4	0	0.0981	2	0.1667
169	-2 0 2 1 2 -1 1 0 -1 1 1 0 -2 -1 -1 1 -1 1 0 -1 0	2	2	3	0
170	-2 0 2 2 -2 4 2 0 -2 1 1 0 -1 1 -2 -1 -1 0 0 -2 0	2	2.0981	3	0.25
171	-2 0 2 2 1 2 -1 1 -1 1 0 -1 -1 -1 -1 0 0 0 0 -2 0	0	0.4158	1	0.2358
172	-2 1 -1 0 -1 -1 4 4 -1 0 1 1 2 -1 1 -2 -2 0 1 1 -4	3	3	4	0
173	-2 1 -1 0 0 0 4 3 -1 0 1 1 2 -1 1 -2 -2 0 1 0 -3	3	3	4	0
174	-2 1 -1 1 -2 -1 4 4 -1 0 1 1 1 0 1 -2 -2 0 1 1 -4	3	3	4	0
175	-2 1 -1 1 -1 0 4 3 -1 0 1 1 1 0 1 -2 -2 0 1 0 -3	3	3	4	0
176	-2 1 -1 1 0 1 4 3 -1 0 1 1 1 -1 0 -2 -2 0 1 -1 -3	2	2	3	0
177	-2 1 -1 1 1 1 2 2 -1 0 1 1 0 -2 0 0 0 0 1 -2 -2	2	2	3	0
178	-2 1 -1 2 0 2 4 3 -2 0 1 1 0 -1 -1 -2 -2 1 1 -2 -2	2	2	3	0
179	-2 1 -1 2 1 1 2 1 -1 0 1 1 -1 -2 0 0 0 0 1 -2 -1	2	2	3	0
180	-2 1 -1 3 1 3 4 3 -3 0 1 1 -1 -2 -2 -2 -2 2 1 -3 -1	2	2	3	0
181	-2 1 1 0 0 0 0 0 0 1 -1 1 -1 -1 -2 1 1 -1 0 0 0	1	1	2	0
182	-2 1 1 1 0 1 0 0 0 1 -1 1 -1 -1 -2 1 1 -1 0 -1 0	1	1	2	0
183	-2 1 1 1 1 2 0 0 0 1 -1 1 -1 -1 -2 1 1 -1 0 -2 0	1	1	2	0
184	-2 1 1 2 2 3 0 0 0 1 -1 1 -1 -1 -2 1 1 -1 0 -4 0	1	1	2	0
185	-2 1 1 3 1 3 2 2 -3 0 0 0 -1 -1 -1 -1 -1 1 0 -4 0	1	1	2	0
186	-2 1 2 -1 -1 -1 1 2 -1 0 0 0 2 0 2 -1 -1 -1 -1 2 -1	3	3	4	0
187	-2 1 2 -1 -1 0 1 1 -1 0 0 0 2 0 2 -1 -1 -1 -1 1 0	3	3	4	0
188	-2 1 2 0 0 0 0 1 -1 1 0 -1 1 0 1 -1 -1 0 -1 0 0	1	1.1739	2	0.2319
189	-2 1 2 0 0 0 1 1 0 0 -1 -1 2 -1 1 0 0 -1 0 0 -1	2	2	3	0
190	-2 1 2 1 -1 0 1 1 0 0 -1 -1 1 0 1 0 0 -1 0 0 -1	2	2	3	0
191	-2 1 2 1 0 1 0 1 -1 1 0 -1 0 0 0 -1 -1 0 -1 -1 0	0	0.3808	1	0.2312
192	-2 1 2 1 1 0 2 0 2 0 -1 -1 0 0 -1 0 -1 -1 0 -1 -2	0	0.0521	1	0.111

193	-2 1 2 1 1 1 1 1 0 0 0 0 1 -1 1 -1 0 -2 -1 -2 -1	0	0.3632	2	0.2355
194	-2 1 2 1 2 2 1 3 -2 0 0 0 1 -2 0 -1 -1 0 -1 -3 -1	1	1	2	0
**195**	**-2 1 2 2 -2 2 2 2 1 1 -1 -1 -1 1 -1 -1 -1 -1 0 -1 -2**	**0**	**0.5**	**1**	**0.25**
**196**	**-2 1 2 2 -1 2 1 1 0 1 -1 -1 -1 0 -1 0 0 0 0 -2 -1**	**0**	**0.4158**	**1**	**0.2358**
197	-2 1 2 2 -1 2 2 2 0 1 -1 -1 -1 1 -1 -1 -1 -1 0 -1 -2	0	0.2249	1	0.2161
198	-2 1 2 2 -1 2 2 2 1 0 -1 -1 0 -1 -1 0 0 -1 0 -2 -3	0	0.0295	1	0.094
199	-2 1 2 2 0 2 1 0 1 1 -1 -1 -1 -1 -1 0 1 -1 0 -2 -1	0	0.3371	1	0.2098
200	-2 1 2 2 1 2 0 0 0 1 -1 -1 -1 -1 -1 0 1 -1 0 -2 0	0	0.3094	1	0.2253
201	-2 1 2 2 1 2 1 2 -2 0 0 0 0 -1 0 -1 -1 0 -1 -3 0	1	1	2	0
202	-2 1 2 2 1 3 0 0 0 1 -1 0 -1 -1 -2 1 1 -1 0 -3 0	1	1	2	0
203	-2 1 2 2 1 3 1 1 -1 0 -1 -1 0 -1 -1 0 0 -1 0 -3 -1	0	0.0386	1	0.1142
204	-2 1 3 0 -1 -1 0 0 0 0 -1 -1 2 0 2 0 1 -2 -1 1 0	3	3	4	0
205	-2 1 3 0 -1 -1 0 1 -1 0 -1 -1 2 0 2 0 0 -1 -1 1 0	3	3	4	0
206	-2 1 3 0 0 0 -1 1 -1 0 -1 -1 2 0 1 0 0 -1 -1 0 0	2	2	3	0
207	-2 1 3 0 0 0 0 -1 1 1 0 -1 -1 0 -2 1 0 0 -1 0 0	1	1	2	0
208	-2 1 3 0 0 0 0 0 0 0 -1 -1 2 -1 1 0 1 -2 -1 0 0	2	2	3	0
209	-2 1 3 0 0 0 0 0 0 1 0 -1 -1 0 -2 1 -1 1 -1 0 0	1	1	2	0
210	-2 1 3 0 0 0 0 1 -1 0 -1 -1 2 -1 1 0 0 -1 -1 0 0	2	2	3	0
211	-2 1 3 0 0 0 2 -1 1 0 -1 -1 0 1 -2 0 0 0 -1 0 -1	1	1	2	0
212	-2 1 3 0 0 0 2 0 2 0 -1 -1 0 1 -2 0 -1 -1 -1 0 -2	0	0	1	0
213	-2 1 3 0 1 -1 0 0 0 1 0 -1 -1 -1 -1 1 -1 1 -1 0 0	1	1	2	0
214	-2 1 3 0 1 -1 2 -1 1 0 -1 -1 0 0 -1 0 0 0 -1 0 -1	1	1	2	0
215	-2 1 3 1 0 1 -1 1 -1 0 -1 -1 1 0 0 0 0 -1 -1 -1 0	1	1	2	0
216	-2 1 3 1 2 -1 4 -2 2 -1 -1 -1 0 0 -1 -1 0 -1 -1 -1 -2	0	0.1449	2	0.2126
217	-2 1 3 2 1 -1 4 -2 3 0 -1 -1 0 0 0 -2 1 -2 -1 -1 -2	1	1	2	0
218	-2 1 3 2 1 2 -2 3 -3 2 0 -1 -2 -2 -1 0 -1 1 -1 -2 0	1	1.0808	2	0.1372
219	-2 1 3 2 1 3 0 0 0 0 -1 -1 0 -1 -1 0 1 -2 -1 -3 0	0	0	1	0
220	-2 1 3 4 -1 4 2 2 -1 0 -1 -1 -2 1 -2 0 -1 -1 -1 -3 -2	0	0	1	0
221	-2 2 -1 2 0 2 6 5 -2 -2 1 1 0 -3 -1 -2 -2 1 0 -2 -4	1	1	3	0
222	-2 2 0 0 0 0 0 0 0 1 -1 1 -1 -1 -1 1 1 -1 0 0 0	1	1	2	0
223	-2 2 0 2 1 3 2 3 -3 1 0 1 -1 -1 -2 -1 -2 2 0 -3 0	2	2.1038	3	0.2203
224	-2 2 0 2 2 2 0 0 0 1 -1 1 -1 -1 -1 1 1 -1 0 -4 0	1	1	2	0
225	-2 2 1 0 0 0 2 2 0 1 -1 -1 1 -1 1 -1 -1 0 0 0 -2	1	1	2	0
226	-2 2 1 2 1 1 1 1 -1 1 -1 -1 -1 -1 0 0 0 0 0 -2 -1	0	0.4347	1	0.2432
227	-2 2 2 -1 -1 0 1 1 0 1 -1 -1 1 0 1 -1 0 -1 -1 1 -1	1	1.4142	2	0.25
228	-2 2 2 0 0 0 1 2 -1 1 -1 -1 1 0 1 -1 -1 0 -1 0 -1	1	1.3371	2	0.25
229	-2 2 2 1 -1 2 1 1 0 1 -1 -1 -1 0 -1 0 0 0 -1 -1 -1	0	0.4365	1	0.25
230	-2 2 2 2 -2 2 2 2 0 0 -1 0 -2 1 -2 0 -1 0 -2 0 -2	0	0.1419	1	0.158
231	-2 2 2 2 -2 2 2 2 0 1 -1 -1 -1 1 -1 -1 -1 -1 0 0 -2	1	1	2	0
**232**	**-2 2 2 2 -2 2 2 2 0 1 -1 -1 -1 1 -1 -1 -1 0 -1 -1 -2**	**0**	**0.5**	**1**	**0.25**
233	-2 2 2 2 2 2 2 2 -1 -1 0 0 1 -2 1 -1 0 -2 -2 -4 -2	0	0	2	0
234	-2 2 2 2 2 2 2 3 -2 -1 0 0 1 -2 1 -1 -1 -1 -2 -4 -2	0	0.0353	2	0.1542
235	-2 2 2 4 1 4 3 3 -4 -1 0 0 -1 -1 -1 -2 -2 1 -2 -5 0	0	0.3331	2	0.1861
236	-2 2 2 6 1 6 5 4 -6 -2 1 0 -2 -1 -2 -3 -3 2 -2 -7 0	1	1	3	0
237	-2 2 3 -1 -1 -1 0 0 0 0 -1 -1 2 0 2 0 1 -2 -2 2 0	3	3	4	0
238	-2 2 3 -1 -1 -1 0 1 -1 0 -1 -1 2 0 2 0 0 -1 -2 2 0	3	3	4	0
239	-2 2 3 -1 -1 -1 1 2 -2 0 -1 -1 2 0 2 -1 -1 0 -2 2 0	3	3	4	0
240	-2 2 3 0 0 0 1 1 0 1 -1 -1 1 -1 1 -1 0 -2 -1 0 -1	1	1	2	0
241	-2 2 3 1 -1 1 1 1 0 1 -1 -1 0 0 0 -1 0 -2 -1 0 -1	1	1	2	0
242	-2 2 3 1 -1 1 1 2 -1 1 -1 -1 0 0 0 -1 -1 -1 -1 0 -1	1	1	2	0

243	-2 2 3 1 0 -1 3 -1 3 1 -1 -1 1 1 0 -2 0 -2 -1 0 -2	2	2	3	0
244	-2 2 3 2 1 2 0 0 0 0 -1 -1 0 -1 0 0 1 -2 -2 -3 0	0	0	1	0
245	-2 2 3 3 -2 3 1 1 0 0 -2 -2 -1 1 -1 0 1 -1 -1 -2 -1	1	1	2	0
246	-2 2 3 3 -1 3 1 1 -1 0 -2 -2 -1 1 -1 0 1 -1 -1 -2 -1	1	1	2	0
247	-2 2 3 3 -1 3 1 1 0 1 -1 -1 -2 0 -2 1 0 -1 -1 -2 -1	1	1	2	0
248	-2 2 3 4 -1 4 3 2 -2 0 -1 -1 -2 1 -2 -1 -1 0 -2 -3 -2	0	0	1	0
249	-2 2 4 0 0 0 0 2 -2 1 -1 -2 1 -1 2 -1 -1 0 -2 0 0	2	2	3	0
250	-2 2 4 2 0 2 0 2 -2 1 -1 -2 -1 -1 0 -1 -1 0 -2 -2 0	0	0	1	0
251	-2 2 4 2 4 -2 4 -2 2 -1 -1 -1 -1 -1 -1 -1 -1 -1 -2 -2 -2	0	0	2	0
252	-2 3 1 0 0 0 4 3 -2 0 -1 -1 2 -1 1 -2 -2 1 -1 0 -2	2	2	3	0
253	-2 3 1 1 -1 0 4 3 -2 0 -1 -1 1 0 1 -2 -2 1 -1 0 -2	2	2	3	0
254	-2 3 2 0 -1 1 3 3 -2 0 -2 -2 1 0 1 -1 -1 1 -1 0 -2	2	2	3	0
255	-2 3 2 0 0 0 3 3 -1 0 -2 -2 1 -2 2 -1 -1 1 -1 0 -2	2	2	3	0
256	-2 3 2 0 0 0 3 3 -1 1 -1 -1 2 -1 1 -2 -2 0 -1 0 -2	2	2	3	0
257	-2 3 3 0 -1 1 2 3 -1 1 -2 -2 1 0 1 -1 -1 0 -1 0 -2	2	2	3	0
258	-2 3 3 0 0 0 2 3 -2 1 -1 -1 2 -1 1 -2 -2 0 -2 0 -1	2	2	3	0
259	-2 3 3 0 0 0 2 3 -1 1 -2 -2 1 -1 2 -1 -1 0 -1 0 -2	2	2	3	0
260	-2 3 4 2 0 2 1 1 0 1 -2 -2 -1 -1 0 -1 1 -2 -2 -2 -1	0	0	1	0
261	-2 3 4 3 3 -3 4 1 3 -1 -1 -1 -2 -2 0 -1 -2 0 -3 0 -4	0	0.0274	2	0.1577
262	-2 3 5 0 1 1 2 5 -3 0 -1 -1 2 -3 1 -2 -2 -1 -3 -1 -2	1	1	3	0
263	-2 4 4 -2 -2 -2 1 3 -3 -1 -2 -1 3 0 3 -1 -1 0 -4 4 0	4	4.2275	6	0.1741
264	-2 4 4 0 0 0 4 2 2 -1 -1 -1 1 1 -3 -1 -3 1 -4 0 -4	0	0	2	0
265	-2 4 4 2 2 -2 4 2 2 -1 -1 -1 -1 -1 -1 -1 -3 1 -4 0 -4	0	0	2	0
266	-2 4 4 2 2 2 0 0 0 -1 -2 -1 1 -2 1 0 2 -3 -4 -4 0	0	0	2	0
267	-2 4 4 2 2 2 0 2 -2 -1 -2 -1 1 -2 1 0 0 -1 -4 -4 0	0	0.027	2	0.1235
268	-2 4 4 2 2 2 2 2 0 -1 -1 -1 1 -3 1 -1 1 -3 -4 -4 -2	0	0	2	0
269	-2 4 4 2 2 2 2 4 -2 -1 -1 -1 1 -3 1 -1 -1 -1 -4 -4 -2	0	0	2	0
270	-2 4 4 4 -2 4 4 4 -2 1 -2 -2 -2 1 -2 -2 -2 1 -3 -3 -3	0	0	1	0
271	-2 4 6 2 -2 2 2 4 -4 1 -1 -2 1 -1 0 -3 -3 0 -4 -2 0	1	1	3	0
272	-2 4 6 2 0 2 -2 2 -2 1 -1 -2 -1 -3 0 1 1 -2 -4 -2 0	1	1	3	0
273	-2 5 5 0 0 0 4 3 1 -1 -1 -2 1 1 -3 -1 -4 2 -5 0 -4	0	0	2	0
274	-2 5 5 1 1 -1 4 3 1 -1 -1 -2 0 0 -2 -1 -4 2 -5 0 -4	0	0	2	0
275	-2 5 5 5 0 5 5 5 -5 -1 -1 -2 -1 -1 -2 -4 -4 2 -5 -5 0	0	0.1545	2	0.1534
276	-2 6 4 -2 -2 2 2 2 0 -1 -2 -3 1 -2 1 1 0 -1 -4 0 -2	1	1	3	0
277	-1 -1 -1 0 0 0 1 1 -1 0 1 1 1 -1 0 0 0 0 2 0 -1	2	2.125	3	0.2098
278	-1 -1 0 -1 1 -1 0 0 0 0 1 1 1 -1 1 0 0 -1 1 1 0	2	2.2071	3	0.25
279	-1 -1 0 0 -1 1 1 2 -1 0 2 2 1 -1 0 -1 -1 -1 1 0 -1	3	3	4	0
280	-1 -1 0 0 0 0 0 0 0 0 1 1 1 -1 1 0 0 -1 1 0 0	2	2	3	0
281	-1 -1 0 0 0 0 0 1 -1 0 1 1 1 -1 0 0 0 0 1 0 0	2	2	3	0
282	-1 -1 0 0 0 0 1 0 1 0 1 1 0 1 -1 0 -1 -1 1 0 -1	1	1	2	0
283	-1 -1 0 0 0 0 1 1 -1 0 2 2 1 -2 2 -1 -1 -1 1 0 0	4	4	5	0
284	-1 -1 0 0 0 0 1 1 0 -1 1 1 2 -1 1 0 0 -1 1 0 -1	3	3	4	0
285	-1 -1 0 0 0 0 1 1 0 0 1 1 1 -1 0 -1 0 -1 1 0 -1	1	1	2	0
286	-1 -1 0 0 0 0 1 2 -1 0 2 2 1 -2 1 -1 -1 -1 1 0 -1	3	3	4	0
287	-1 -1 0 0 1 -1 1 -1 0 0 1 1 0 0 0 0 0 0 1 0 0	2	2.2071	3	0.25
288	-1 -1 0 0 1 -1 1 0 1 0 1 1 0 0 0 0 -1 -1 1 0 -1	1	1.299	2	0.25
289	-1 -1 0 0 1 1 1 2 -1 -1 1 1 2 -2 0 0 0 0 1 -1 -1	3	3	4	0
290	-1 -1 0 0 1 1 2 2 0 -1 1 1 2 -2 0 -1 0 -1 1 -1 -2	2	2	3	0
291	-1 -1 0 0 2 2 5 5 0 -2 2 2 3 -4 0 -3 -1 -2 1 -2 -5	2	2	4	0
292	-1 -1 0 1 -1 0 1 1 0 -1 1 1 1 0 1 0 0 -1 1 0 -1	3	3	4	0

293	-1 -1 0 1 -1 1 1 1 -1 0 2 2 0 -1 1 -1 -1 -1 1 -1 0	3	3	4	0
294	-1 -1 0 1 -1 1 1 2 -2 0 2 2 0 -1 0 -1 -1 0 1 -1 0	3	3	4	0
295	-1 -1 0 1 0 1 2 2 0 -1 1 1 1 -2 -1 -1 0 -1 1 -1 -2	1	1	2	0
296	-1 -1 0 1 1 0 1 -1 0 0 1 1 -1 0 -1 0 0 0 1 -1 0	1	1.1547	2	0.1959
297	-1 -1 0 1 1 0 2 0 2 -1 1 1 0 0 -1 0 -1 -1 1 -1 -2	1	1	2	0
298	-1 -1 0 1 1 2 1 2 -1 -1 1 1 1 -2 -1 0 0 0 1 -2 -1	2	2	3	0
299	-1 -1 0 1 2 -1 3 -2 1 -1 1 1 0 0 0 -1 0 -1 1 -1 -1	2	2	3	0
300	-1 -1 0 2 -1 1 1 1 -1 0 1 1 -1 0 -1 0 0 0 1 -1 -1	1	1.3913	2	0.2371
301	-1 -1 0 2 1 3 1 0 1 -1 1 2 0 -2 -2 0 2 -2 1 -3 -1	2	2	3	0
302	-1 -1 0 3 -1 3 0 0 0 -1 1 1 -1 0 -1 1 0 -2 1 -3 0	1	1	2	0
303	-1 -1 0 4 -1 3 2 1 -1 -1 1 2 -2 0 -2 0 0 0 1 -3 -1	2	2	3	0
304	-1 -1 0 4 0 4 0 0 0 -2 1 2 -1 -1 -2 1 1 -2 1 -4 0	1	1	3	0
305	-1 -1 0 4 0 4 1 1 -1 -2 1 2 -1 -1 -2 0 0 -1 1 -4 0	1	1.0466	3	0.1797
306	-1 -1 0 5 5 0 5 -5 2 -2 2 2 -3 -1 -2 -2 1 -2 1 -5 0	2	2.0009	4	0.0451
307	-1 -1 1 0 2 -2 3 0 3 -1 1 2 1 0 0 -1 -2 -3 0 0 -3	1	1.0334	3	0.2183
308	-1 -1 1 1 1 -1 3 0 3 -1 1 1 0 0 0 -1 -1 -2 0 0 -3	1	1	2	0
309	-1 -1 1 1 1 0 3 -1 2 -1 1 1 0 1 -1 -1 -1 -2 0 -1 -2	1	1	2	0
310	-1 -1 1 1 2 -1 2 0 2 -1 1 1 0 0 0 0 -2 -2 0 -1 -2	0	0.4142	2	0.25
311	-1 -1 1 1 2 -1 3 -1 2 -1 1 1 0 0 0 -1 -1 -2 0 -1 -2	1	1	2	0
312	-1 -1 1 2 0 2 1 1 0 -1 1 1 0 -2 -2 0 1 -1 0 -2 -1	0	0.1514	2	0.1891
313	-1 -1 1 2 0 2 3 3 0 -2 1 1 1 -3 -2 -1 0 -1 0 -2 -3	0	0	2	0
314	-1 -1 1 2 0 2 5 5 1 -3 1 1 2 -4 -2 -2 -1 -2 0 -2 -5	0	0	2	0
315	-1 -1 1 2 1 3 1 1 0 -1 1 1 0 -2 -2 0 1 -1 0 -3 -1	1	1	2	0
316	-1 -1 1 2 1 3 3 3 0 -2 1 1 1 -3 -2 -1 0 -1 0 -3 -3	0	0.0173	2	0.2023
317	-1 -1 1 2 1 3 5 5 1 -3 1 1 2 -4 -2 -2 -1 -2 0 -3 -5	0	0	2	0
318	-1 -1 1 2 2 0 4 -1 3 -2 1 1 0 0 -1 -1 -1 -2 0 -2 -3	0	0.1794	2	0.2091
319	-1 -1 1 2 2 4 3 3 -1 -2 1 1 1 -3 -2 -1 0 -1 0 -4 -3	0	0.0609	2	0.2326
320	-1 -1 1 2 3 5 5 5 -1 -3 1 1 2 -4 -2 -2 -1 -2 0 -5 -5	0	0	2	0
321	-1 -1 1 3 0 3 4 4 1 -3 1 1 1 -3 -2 -1 -1 -2 0 -3 -4	0	0	2	0
322	-1 -1 1 3 1 4 2 2 -1 -2 1 1 0 -2 -2 0 0 -1 0 -4 -2	0	0.0838	2	0.2363
323	-1 -1 1 4 0 4 1 -1 -1 -1 1 1 -2 -1 -2 1 1 -1 0 -4 0	1	1	2	0
324	-1 -1 1 4 0 4 2 0 -2 -1 1 1 -2 -1 -2 0 0 0 0 -4 0	0	0.5298	2	0.218
325	-1 -1 1 4 0 4 2 1 -1 -2 1 1 -1 -1 -2 0 0 -1 0 -4 -1	0	0.1763	2	0.2174
326	-1 -1 1 4 0 4 3 3 0 -3 1 1 0 -2 -2 0 -1 -2 0 -4 -3	0	0	2	0
327	-1 -1 1 4 1 5 3 3 -1 -3 1 1 0 -2 -2 0 -1 -2 0 -5 -3	0	0	2	0
328	-1 -1 1 5 0 5 0 0 0 -2 1 1 -2 -1 -2 2 0 -3 0 -5 0	0	0	2	0
329	-1 -1 1 5 0 5 2 0 -2 -2 1 1 -2 -1 -2 0 0 -1 0 -5 0	0	0.0551	2	0.1467
330	-1 -1 1 5 0 5 2 2 0 -3 1 1 -1 -1 -2 1 -1 -3 0 -5 -2	0	0	2	0
331	-1 -1 1 5 0 5 3 2 -1 -3 1 1 -1 -1 -2 0 -1 -2 0 -5 -2	0	0	2	0
332	-1 -1 1 6 0 6 1 1 0 -3 1 1 -2 -1 -2 2 -1 -4 0 -6 -1	0	0	2	0
333	-1 -1 1 6 0 6 3 1 -2 -3 1 1 -2 -1 -2 0 -1 -2 0 -6 -1	0	0	2	0
334	-1 0 -1 0 0 0 2 1 -1 0 1 1 1 -1 1 -1 -1 0 1 0 -1	2	2	3	0
335	-1 0 -1 1 -1 0 4 3 -1 -1 2 1 1 0 1 -2 -2 0 1 0 -3	3	3	4	0
336	-1 0 -1 1 -1 2 1 1 -2 0 2 2 -1 -1 -1 0 0 0 1 -1 0	3	3	4	0
337	-1 0 -1 3 -1 3 3 3 -4 0 2 2 -1 -1 -2 -2 -2 2 1 -3 0	3	3	4	0
338	-1 0 0 0 0 0 0 0 0 0 0 0 0 0 0 0 0 0 0 0 0	0	0	0	0
339	-1 0 1 -1 -1 -1 2 2 -1 -1 1 0 2 0 2 -1 -1 -1 0 2 -2	3	3	4	0
340	-1 0 1 -1 0 -1 0 0 0 0 0 0 0 0 0 1 0 0 0 1 0	1	1.2071	2	0.25
341	-1 0 1 0 -1 1 1 1 -1 0 1 1 1 0 1 -1 -1 -1 0 0 0	3	3	4	0
342	-1 0 1 0 0 0 0 0 0 0 0 0 -1 0 -1 1 0 0 0 0 0	0	0	1	0

343	-1 0 1 0 0 0 0 1 -1 0 0 0 1 -1 0 0 0 0 0 0 0	1	1	2	0
344	-1 0 1 0 0 0 1 0 1 0 0 0 0 1 -1 0 -1 -1 0 0 -1	0	0	1	0
345	-1 0 1 0 0 0 1 1 -1 0 1 1 1 -1 2 -1 -1 -1 0 0 0	3	3	4	0
346	-1 0 1 0 0 0 1 1 0 0 1 1 1 -1 1 -1 0 -2 0 0 -1	1	1	3	0
347	-1 0 1 0 0 0 1 2 -1 0 1 1 1 -1 1 -1 -1 -1 0 0 -1	2	2	3	0
348	-1 0 1 0 1 -1 1 -1 0 0 0 0 0 0 0 0 0 0 0 0 0	1	1.299	2	0.25
**349**	**-1 0 1 0 1 -1 1 1 2 0 0 0 0 0 0 0 -1 -1 0 0 -2**	**0**	**0.4142**	**1**	**0.25**
350	-1 0 1 0 1 1 1 3 -2 0 1 1 1 -2 0 -1 -1 0 0 -1 -1	2	2	3	0
351	-1 0 1 1 -1 1 1 2 -1 0 1 1 0 0 0 -1 -1 -1 0 0 -1	2	2	3	0
352	-1 0 1 1 -1 2 1 1 -1 0 1 1 0 0 0 -1 -1 -1 0 -1 0	2	2	3	0
353	-1 0 1 1 0 1 1 1 -1 0 1 1 0 -1 1 -1 -1 -1 0 -1 0	2	2	3	0
354	-1 0 1 1 0 1 2 2 0 -1 0 0 1 -2 -1 -1 0 -1 0 -1 -2	0	0	1	0
355	-1 0 1 1 1 0 2 0 2 -1 0 0 0 0 -1 0 -1 -1 0 -1 -2	0	0	1	0
356	-1 0 1 1 1 2 0 0 0 0 0 0 0 -1 -1 0 1 -1 0 -2 0	0	0.1784	1	0.2185
357	-1 0 1 1 1 2 1 3 -3 0 1 1 0 -2 -1 -1 -1 1 0 -2 0	2	2	3	0
358	-1 0 1 1 1 2 2 2 0 -1 0 0 1 -2 -1 -1 0 -1 0 -2 -2	0	0	1	0
359	-1 0 1 1 2 3 2 2 -1 -1 0 0 1 -2 -1 -1 0 -1 0 -3 -2	0	0	1	0
360	-1 0 1 2 1 1 2 1 -1 -1 1 0 0 -1 1 -1 -1 -1 0 -2 -1	1	1	2	0
361	-1 0 1 3 -3 4 3 2 -1 0 1 1 -2 2 -2 -1 -2 -1 0 -1 -2	2	2	3	0
362	-1 0 1 3 -1 3 2 1 -1 -1 1 0 -1 1 -1 -1 -1 -1 0 -2 -1	1	1	2	0
363	-1 0 1 3 -1 3 2 1 -1 0 1 1 -2 0 -2 0 0 0 0 -2 -1	2	2	3	0
364	-1 0 1 3 0 3 3 0 -3 0 1 0 -2 -1 -1 -1 -1 1 0 -3 0	1	1	2	0
365	-1 0 1 7 2 5 5 2 -5 -3 2 0 -3 -2 -1 -2 -2 1 0 -7 0	1	1	3	0
366	-1 1 -1 -3 3 1 -1 -2 -1 2 0 2 2 -1 -2 -1 0 1 1 0 2	5	5	6	0
367	-1 1 -1 -3 3 1 0 -1 -1 2 0 2 2 -1 -2 -2 -1 1 1 0 1	4	4	5	0
368	-1 1 -1 -2 2 1 -2 -1 1 2 0 2 1 -1 -2 0 0 0 1 0 1	4	4	5	0
369	-1 1 -1 -2 2 1 -1 -1 0 2 0 2 1 -1 -2 -1 0 1 1 0 1	4	4	5	0
370	-1 1 -1 -2 2 1 0 0 0 2 0 2 1 -1 -2 -2 -1 1 1 0 0	3	3	4	0
371	-1 1 -1 -1 2 1 -1 -1 1 2 0 2 0 -1 -2 -1 0 1 1 -1 0	3	3	4	0
372	-1 1 -1 -1 2 1 0 0 0 2 0 2 0 -1 -2 -2 -1 2 1 -1 0	3	3	4	0
373	-1 1 -1 0 0 0 -3 -1 2 2 0 2 -1 2 -1 1 -1 -2 1 0 1	4	4	5	0
374	-1 1 -1 1 0 1 4 4 -1 -2 1 1 1 -3 -1 -1 -1 0 0 -1 -4	0	0	2	0
375	-1 1 -1 1 1 2 4 4 -1 -2 1 1 1 -3 -1 -1 -1 0 0 -2 -4	0	0.0942	2	0.1859
376	-1 1 -1 1 2 3 4 4 -2 -2 1 1 1 -3 -1 -1 -1 0 0 -3 -4	0	0.2038	2	0.1945
377	-1 1 -1 2 0 2 4 3 -1 -2 1 1 0 -2 -1 -1 -1 0 0 -2 -3	0	0.1794	2	0.2091
378	-1 1 -1 2 0 2 6 5 -2 -3 2 1 0 -3 -1 -2 -2 1 0 -2 -4	1	1	3	0
379	-1 1 0 0 0 0 3 3 -1 0 1 1 2 -1 1 -2 -2 0 0 0 -2	3	3	4	0
**380**	**-1 1 0 0 2 2 1 1 0 0 -1 1 1 -1 -1 -1 0 -1 0 -2 -1**	**0**	**0.2071**	**1**	**0.25**
381	-1 1 0 1 -1 0 3 3 -1 0 1 1 1 0 1 -2 -2 0 0 0 -2	3	3	4	0
382	-1 1 0 1 0 1 1 1 -1 0 1 1 -1 -2 0 0 0 0 0 -1 0	1	1.0989	3	0.1682
383	-1 1 0 1 0 1 3 3 -1 0 1 1 1 -1 0 -2 -2 0 0 -1 -2	2	2	3	0
384	-1 1 0 1 1 1 1 1 -1 0 0 0 -1 -1 -1 0 -1 1 0 -1 0	0	0.25	1	0.25
385	-1 1 0 1 1 1 1 2 -1 0 1 1 0 -2 0 0 0 0 0 -2 -1	2	2	3	0
386	-1 1 0 2 1 1 1 1 -1 0 1 1 -1 -2 0 0 0 0 0 -2 0	2	2	3	0
387	-1 1 1 -1 -1 -1 2 2 -2 -1 0 0 2 0 2 -1 -1 0 -1 2 -1	3	3	4	0
388	-1 1 1 -1 -1 1 0 1 -1 -1 1 0 -1 -1 -1 2 0 1 -1 0 0	1	1.095	3	0.166
389	-1 1 1 0 0 0 1 1 0 0 -1 -1 1 -1 1 0 0 0 0 0 -1	1	1	2	0
390	-1 1 1 0 0 0 2 1 -1 -1 -1 -1 2 -1 1 0 0 0 0 0 -1	2	2	3	0
391	-1 1 1 0 1 -1 1 1 0 -1 0 1 -1 -1 -1 1 -1 1 -1 0 -1	1	1	2	0
392	-1 1 1 0 5 -5 2 -1 1 0 0 2 1 -1 2 -1 -3 -3 0 0 -1	3	3	5	0

393	-1 1 1 1 1 -1 2 1 1 -1 0 1 -1 -1 -1 0 -1 1 -1 0 -2	1	1	2	0
394	-1 1 1 1 1 1 1 1 -1 -1 0 0 1 -1 1 0 0 -1 -1 -2 -1	0	0.25	2	0.25
395	-1 1 1 1 2 -1 2 -1 1 0 0 0 0 0 0 -1 -1 -1 0 -1 -1	1	1	2	0
396	-1 1 1 1 2 2 1 3 -2 -1 0 1 1 -2 0 -1 -1 0 -1 -3 -1	1	1	2	0
397	-1 1 1 1 2 2 2 2 -1 -1 0 0 1 -2 0 -1 0 -1 -1 -3 -2	0	0	1	0
398	-1 1 1 2 -1 1 2 1 -1 -1 -1 -1 0 0 0 0 0 0 0 -1 -1	1	1	2	0
399	-1 1 1 2 1 2 1 1 -1 -1 0 0 0 -1 0 0 0 -1 -1 -3 -1	0	0	1	0
400	-1 1 1 2 1 2 1 2 -2 -1 0 1 0 -1 0 -1 -1 0 -1 -3 0	1	1	2	0
401	-1 1 1 2 1 2 2 2 -2 -1 0 0 0 -1 0 -1 -1 0 -1 -3 -1	0	0	1	0
402	-1 1 1 2 2 2 3 3 -2 -2 1 0 1 -2 1 -1 -1 -1 -1 -4 -3	0	0	2	0
403	-1 1 1 5 1 4 4 2 -4 -2 1 0 -2 -1 -1 -2 -2 1 -1 -5 0	0	0.2731	2	0.1695
404	-1 1 2 -1 1 -2 1 0 1 -1 -1 0 0 0 0 1 -1 -1 -1 1 -1	1	1	2	0
405	-1 1 2 0 0 0 0 0 0 0 -1 -1 1 0 1 0 1 -1 -1 0 0	1	1	2	0
406	-1 1 2 0 0 0 1 0 1 -1 -1 0 -1 1 -2 1 -1 -1 -1 0 -1	0	0	1	0
407	-1 1 2 0 0 0 1 1 -1 -1 -1 -1 2 -1 1 0 0 0 -1 0 0	2	2	3	0
408	-1 1 2 0 0 0 1 1 0 0 0 0 1 -1 1 -1 0 -2 -1 0 -1	0	0.2071	2	0.25
409	-1 1 2 0 1 -1 1 0 1 -1 -1 0 -1 0 -1 1 -1 -1 -1 0 -1	0	0	1	0
410	-1 1 2 1 0 1 1 1 0 -1 -1 0 1 -2 -1 -1 1 -2 -1 -1 -1	0	0	1	0
411	-1 1 2 1 0 1 1 2 -1 -1 -1 0 1 -2 -1 -1 0 -1 -1 -1 -1	0	0	1	0
412	-1 1 2 1 1 0 1 -1 1 0 0 0 -1 0 -2 0 0 0 -1 -1 0	0	0.2035	2	0.2107
413	-1 1 2 1 2 -1 2 -1 1 -1 -1 0 -1 0 -1 0 -1 -1 -1 -1 -1	0	0	1	0
414	-1 1 2 1 2 3 1 1 0 -1 -1 0 1 -2 -1 -1 1 -2 -1 -3 -1	0	0	1	0
415	-1 1 2 1 2 3 1 2 -1 -1 -1 0 1 -2 -1 -1 0 -1 -1 -3 -1	0	0	1	0
416	-1 1 2 2 0 2 0 2 -2 1 0 -1 -1 -1 -1 -1 -1 1 -1 -2 0	0	0.2361	1	0.25
417	-1 2 1 -1 1 1 4 4 -1 -2 0 -1 2 -3 0 -1 -1 0 -1 0 -4	0	0.0437	2	0.1724
418	-1 2 1 0 0 0 2 2 -1 0 -1 -1 1 -1 1 -1 -1 1 -1 0 -1	1	1.0097	2	0.0985
419	-1 2 1 0 0 0 3 3 -1 -1 -1 -1 1 -2 1 -1 -1 1 -1 0 -2	1	1	2	0
420	-1 2 2 -1 -2 2 1 0 1 -1 0 -1 -1 0 -1 1 0 -1 -2 0 -1	0	0	1	0
421	-1 2 2 -1 -1 -1 1 2 -2 -1 -1 0 2 0 2 -1 -1 0 -2 2 0	3	3	4	0
422	-1 2 2 0 -2 2 1 1 1 -1 0 -1 -1 0 -1 1 0 -1 -2 0 -2	0	0	1	0
423	-1 2 2 1 0 1 1 1 0 0 -1 -1 0 -1 0 0 0 -1 -1 -1 -1	0	0.069	1	0.157
424	-1 2 2 1 0 1 2 2 -1 0 -1 -1 0 -1 0 -1 -1 0 -1 -1 -1	0	0.0833	1	0.2233
425	-1 2 2 1 2 2 1 1 0 -1 -1 0 1 -2 0 -1 1 -2 -2 -3 -1	0	0	1	0
426	-1 2 2 1 2 3 3 4 -3 -1 0 -1 1 -2 -1 -2 -2 1 -2 -3 -2	0	0	1	0
427	-1 2 2 2 -1 2 2 2 -1 0 -1 -1 -1 0 -1 -1 -1 0 -1 -1 -1	0	0	1	0
428	-1 2 2 2 1 2 0 0 0 -1 -1 0 0 -1 0 0 1 -2 -2 -3 0	0	0	1	0
429	-1 2 2 2 1 2 0 1 -1 -1 -1 0 0 -1 0 0 0 -1 -2 -3 0	0	0	1	0
430	-1 2 2 3 2 1 0 0 0 -1 0 -1 -1 -2 1 1 0 -2 -2 -3 0	0	0	1	0
431	-1 2 2 3 4 -1 2 -2 0 -1 0 -1 -1 -2 1 -1 0 -1 -2 -3 0	0	0	1	0
432	-1 2 3 0 0 0 0 1 -1 0 -1 -1 1 -1 1 0 0 -1 -2 0 0	1	1	2	0
433	-1 2 3 0 1 -1 0 0 0 1 0 -1 -1 -1 -1 1 -2 1 -2 0 0	1	1	2	0
434	-1 2 3 1 2 -2 0 0 0 1 0 -1 -2 -2 0 2 -2 1 -2 0 0	2	2	3	0
435	-1 3 3 0 -3 3 1 1 1 -1 -1 -1 -2 1 -2 1 -1 -1 -3 0 -1	0	0	1	0
436	-1 3 3 0 0 0 2 3 -3 0 -1 -1 2 -1 1 -2 -2 1 -3 0 0	2	2	3	0
437	-1 3 3 1 2 3 2 4 -3 -1 -1 -1 1 -2 -1 -2 -2 1 -3 -3 -1	0	0	1	0
438	-1 3 3 2 -2 1 1 1 0 2 -1 -2 -1 0 1 -2 0 -1 -2 -1 -1	1	1	2	0
439	-1 3 3 3 3 -3 4 1 3 -2 -1 0 -2 -2 0 -1 -2 0 -3 0 -4	0	0.0834	2	0.1114
440	-1 3 4 0 -2 2 0 3 -3 0 -2 -2 1 1 0 -1 -1 1 -3 0 0	2	2	3	0
441	-1 3 4 3 -1 4 3 3 -3 -1 -1 -1 -1 -1 -1 -2 -2 0 -3 -3 0	0	0.0001	2	0.0323
442	-1 3 4 3 0 3 3 3 -3 -1 -1 -1 -1 -2 0 -2 -2 0 -3 -3 0	0	0.0083	2	0.0999

443	-1 4 3 1 1 1 3 4 -1 -1 -1 -1 1 -3 1 -1 -1 -1 -3 -2 -3	0	0	2	0
444	-1 4 4 2 -2 1 2 2 -1 2 -2 -2 -1 0 1 -2 0 -1 -3 -1 -2	1	1	2	0
445	-1 4 4 2 3 -2 3 -3 1 2 -2 -2 -2 -1 0 -1 1 1 -3 -2 -1	2	2	3	0
446	-1 4 4 5 4 -7 5 -2 3 2 -2 0 -1 -2 3 -4 0 -3 -3 -2 -3	2	2.0941	4	0.2057
447	-1 4 5 2 -2 2 -1 1 -2 2 -1 -3 -1 -1 1 -1 0 0 -4 -2 1	1	1.1787	3	0.2295
448	-1 5 6 3 -3 3 3 5 -5 1 -1 -2 1 -1 -1 -4 -4 1 -5 -3 0	1	1	3	0
449	-1 6 6 3 -2 3 3 2 -5 2 -2 -2 -2 -2 -1 -1 0 1 -5 -3 0	2	2	4	0
450	-1 6 7 3 -2 3 -1 2 -3 2 -2 -4 -2 -2 1 -1 0 0 -6 -3 1	1	1	3	0
451	0 -2 -2 0 0 0 -1 1 -1 2 2 0 -2 1 -1 0 -2 2 2 0 1	3	3	5	0
452	0 -2 2 0 0 0 0 -2 -2 1 1 0 -1 1 0 1 1 0 0 0 2	4	4	5	0
453	0 -1 -1 -5 5 0 -1 -1 1 4 3 0 1 -2 -1 0 -2 0 1 0 1	6	6	8	0
454	0 -1 -1 -5 5 0 0 0 0 4 3 0 1 -2 -1 -1 -3 1 1 0 0	5	5	7	0
455	0 -1 -1 -1 1 0 -1 -1 0 1 1 -1 0 1 1 1 -1 2 1 0 1	5	5	6	0
456	0 -1 -1 -1 2 1 0 -1 1 2 2 0 -1 -1 -1 1 -1 0 1 -1 0	3	3	4	0
457	0 -1 -1 0 0 0 -1 -1 -1 2 1 0 -2 2 -1 -1 -1 1 1 0 1	2	2	4	0
458	0 -1 -1 0 0 0 -1 -1 0 1 1 -1 -1 2 1 1 -1 2 1 0 1	5	5	6	0
459	0 -1 -1 0 0 0 -1 -1 1 2 1 0 -2 1 -1 0 0 0 1 0 1	2	2	4	0
460	0 -1 -1 0 0 0 0 -1 1 1 1 -1 -1 1 1 1 0 1 1 0 0	4	4	5	0
461	0 -1 -1 0 0 0 0 0 0 1 1 -1 -1 1 1 1 -1 2 1 0 0	4	4	5	0
462	0 -1 -1 0 0 0 0 0 0 2 1 0 -2 1 -1 -1 -1 1 1 0 0	1	1	3	0
463	0 -1 1 0 0 0 2 0 2 -1 1 1 1 1 -1 -1 -1 -2 0 0 -2	1	1	2	0
464	0 -1 1 1 2 3 3 3 -1 -2 1 0 1 -2 -1 -1 -1 -1 0 -3 -3	0	0	1	0
465	0 -1 1 2 0 2 2 2 0 -2 1 0 0 -1 -1 0 -1 -1 0 -2 -2	0	0	1	0
466	0 -1 1 2 1 3 2 2 -1 -2 1 0 0 -1 -1 0 -1 -1 0 -3 -2	0	0	1	0
467	0 0 0 -7 4 -5 -3 2 5 4 2 -1 3 -2 1 0 -2 -1 0 3 -2	7	7	9	0
468	0 0 0 -7 5 -6 -1 -5 -4 4 1 -2 3 -1 2 -2 1 4 0 2 5	10	10	12	0
469	0 0 0 -5 4 -4 0 -3 -3 3 1 -1 2 -1 1 -2 0 3 0 1 3	6	6.182	8	0.1553
470	0 0 0 -4 0 -4 -1 -1 1 3 0 -2 1 1 2 0 0 1 0 4 1	6	6	8	0
471	0 0 0 -4 0 -4 0 0 0 3 0 -2 1 1 2 -1 -1 2 0 4 0	5	5	7	0
472	0 0 0 -4 1 -3 -1 -1 0 2 1 -1 2 -1 1 -1 1 2 0 3 1	6	6	7	0
473	0 0 0 -4 3 -3 -4 -1 3 1 2 2 2 -2 2 2 0 -2 0 3 1	8	8	9	0
474	0 0 0 -4 4 0 -4 -4 -2 2 1 -2 2 -1 1 2 1 2 0 0 6	8	8.0275	10	0.1268
475	0 0 0 -4 4 0 -1 -1 -1 2 2 0 2 -2 -1 -1 -1 1 0 0 1	3	3.0656	5	0.2394
476	0 0 0 -3 -1 -3 -1 1 -1 2 0 -2 1 2 2 0 -2 2 0 4 1	5	5.0883	7	0.2088
477	0 0 0 -3 0 -3 -1 1 1 2 1 -1 1 1 1 0 -1 1 0 3 -1	5	5	6	0
478	0 0 0 -3 1 -3 -1 2 2 2 2 -1 1 1 1 0 -2 0 0 2 -2	5	5	6	0
479	0 0 0 -3 2 -3 0 -2 -2 2 1 -1 1 0 1 -1 0 2 0 1 2	5	5	6	0
480	0 0 0 -3 3 -2 -1 -3 -2 2 1 -2 1 -1 0 0 1 2 0 0 3	5	5	6	0
481	0 0 0 -3 3 -1 -2 -1 1 1 2 1 2 -2 0 0 0 0 0 1 1	5	5	6	0
482	0 0 0 -3 3 -1 0 -1 -1 2 1 -1 1 -1 1 -1 -1 0 0 0 1	3	3	4	0
483	0 0 0 -3 3 -1 0 0 0 2 1 -1 1 -1 1 -1 -2 -1 0 0 0	2	2	3	0
484	0 0 0 -3 3 0 -1 -1 -1 2 2 0 1 -2 1 0 0 0 0 0 2	4	4.1432	6	0.1845
485	0 0 0 -3 3 0 0 0 0 1 1 -1 2 -1 1 -2 -2 0 0 0 0	1	1	3	0
486	0 0 0 -2 -1 -2 -2 2 1 2 2 -1 0 1 1 2 -2 0 0 3 0	6	6	7	0
487	0 0 0 -2 0 -2 -2 2 2 2 2 -1 0 0 1 2 -2 -1 0 2 0	5	5	6	0
488	0 0 0 -2 0 -2 -1 -1 0 2 0 -1 0 1 1 0 0 1 0 2 1	4	4	5	0
489	0 0 0 -2 0 -2 -1 1 1 2 2 -1 0 1 1 1 -2 0 0 2 0	5	5	6	0
490	0 0 0 -2 1 -2 0 -1 -1 2 2 -1 0 1 1 0 -1 1 0 1 1	5	5	6	0
491	0 0 0 -2 1 -1 -2 -1 -1 2 1 -1 0 1 1 1 -1 1 0 1 2	5	5	6	0
492	0 0 0 -2 1 -1 -1 -1 -1 2 2 -1 0 1 1 1 -1 1 0 1 2	6	6	7	0

493	0 0 0 -2 1 -1 0 -5 5 1 -1 -3 1 3 3 -1 1 -2 0 1 0	5	5	7	0
494	0 0 0 -2 1 2 -1 -1 1 2 1 1 0 1 -1 0 -1 -1 0 -1 0	3	3	4	0
495	0 0 0 -2 2 -2 -2 -2 -2 2 0 -1 0 0 1 0 0 1 0 0 2	3	3	4	0
496	0 0 0 -2 2 0 -2 -2 -1 1 1 -1 1 0 1 1 0 1 0 0 3	5	5	6	0
497	0 0 0 -2 2 0 -2 -2 0 0 0 -1 1 -1 1 1 1 1 0 0 2	3	3.0811	4	0.141
498	0 0 0 -2 2 0 -1 -1 -1 1 1 -1 1 -1 -1 0 0 1 0 0 1	2	2	3	0
499	0 0 0 -2 2 0 -1 1 -1 1 3 2 1 0 -2 0 -3 2 0 0 1	5	5	7	0
500	0 0 0 -1 -1 -2 -2 -2 0 2 0 -1 -1 2 1 1 0 1 0 2 2	5	5	6	0
501	0 0 0 -1 -1 -1 -1 1 0 2 1 -1 -1 1 1 2 -1 1 0 2 0	5	5	6	0
502	0 0 0 -1 -1 -1 0 0 0 2 1 -1 -1 1 1 2 -1 1 0 2 0	5	5	6	0
503	0 0 0 -1 -1 2 -1 -1 0 2 1 1 -1 2 -1 1 -1 -1 0 0 1	4	4	5	0
504	0 0 0 -1 0 -1 -2 -2 0 2 0 -1 -1 2 1 1 0 1 0 1 2	5	5	6	0
505	0 0 0 -1 0 -1 0 -1 -1 2 2 -1 -1 1 1 1 -1 1 0 1 1	5	5	6	0
506	0 0 0 -1 0 -1 0 0 0 1 0 -1 0 1 1 0 -1 1 0 1 0	2	2	3	0
507	0 0 0 -1 1 -1 0 -1 -1 1 1 -1 0 0 0 0 0 1 0 0 1	2	2	3	0
508	0 0 0 -1 1 -1 0 0 0 0 1 1 1 -1 0 -1 -1 1 0 1 0	2	2	3	0
509	0 0 0 -1 1 0 0 0 0 1 0 -1 0 0 0 0 -1 1 0 0 0	1	1	2	0
510	0 0 0 0 0 0 0 -1 -1 1 1 -1 -1 1 -1 0 0 1 0 0 1	2	2	3	0
511	0 0 0 0 0 0 0 0 0 -1 0 -1 0 0 1 1 0 -1 0 0 0	0	0	1	0
512	0 0 0 0 0 0 0 0 0 -1 1 1 -1 -1 -1 1 1 -1 0 0 0	1	1	2	0
513	0 1 -1 -5 6 2 -2 -2 2 4 1 1 1 -2 -1 -1 -2 -1 0 -2 0	4	4	6	0
514	0 1 -1 -4 1 4 -1 -1 0 2 0 2 2 -1 -2 -1 1 -2 0 0 1	4	4	5	0
515	0 1 -1 -3 3 1 -3 -3 0 1 0 2 2 -1 -2 1 1 -1 0 0 3	5	5	6	0
516	0 1 -1 -3 4 1 0 -1 1 2 1 1 1 -2 -1 -1 -1 0 0 -1 0	3	3	4	0
517	0 1 -1 -3 4 2 0 -2 2 2 1 1 1 -2 -1 -1 0 -1 0 -2 0	3	3	4	0
518	0 1 -1 -3 4 2 2 2 -2 3 0 1 0 -1 -1 -3 -3 1 0 -2 0	2	2	4	0
519	0 1 -1 -2 1 1 -3 -1 -3 1 0 -1 0 0 0 2 0 2 0 0 4	5	5	6	0
520	0 1 -1 -2 2 -1 0 -3 -3 1 -1 1 1 0 -1 -1 1 2 0 0 3	4	4	5	0
521	0 1 -1 -1 0 1 -1 -1 0 2 0 2 -1 2 -1 1 -1 -2 0 0 1	4	4	5	0
522	0 1 -1 -1 1 -1 0 3 3 1 1 0 0 -1 2 1 -2 -2 0 1 -3	3	3	4	0
523	0 1 -1 -1 1 0 -3 -1 -3 1 0 -1 -1 0 1 2 0 2 0 0 4	5	5	6	0
524	0 1 -1 -1 1 0 -2 -1 1 1 0 2 0 1 -1 1 -1 -2 0 0 1	3	3	4	0
525	0 1 -1 -1 1 0 -1 0 -1 1 0 0 0 0 1 0 -1 0 0 0 1	2	2	3	0
526	0 1 1 -1 1 0 -1 -1 1 1 1 0 0 1 -1 1 -1 -1 -1 0 1	3	3	4	0
527	0 1 1 0 2 2 -1 -2 1 1 0 -2 -1 0 -1 1 0 1 -1 -2 1	2	2	3	0
528	0 1 1 0 5 5 -2 -3 1 2 1 -3 -2 -1 -3 2 -1 2 -1 -5 2	3	3	5	0
**529**	**0 1 1 1 1 -1 1 -1 1 0 0 -1 -1 -1 1 0 0 0 -1 0 -1**	**0**	**0.25**	**1**	**0.25**
530	0 1 1 2 1 -2 1 -1 -1 1 0 1 -1 1 0 -1 -1 0 -1 -1 0	2	2	3	0
531	0 1 1 4 4 -5 3 -3 0 1 0 2 -1 -1 2 -3 0 -2 -1 -3 0	2	2.3249	4	0.1815
532	0 1 1 4 5 -7 2 -5 -3 2 0 3 -2 -1 3 -2 1 0 -1 -2 3	7	7	9	0
533	0 1 1 6 5 -7 4 -5 -1 2 0 3 -2 -1 3 -4 1 -2 -1 -4 1	5	5	7	0
534	0 2 -2 -3 3 2 -1 -1 -1 1 0 2 2 -2 -2 -1 0 0 0 0 1	3	3.0349	4	0.1041
535	0 2 -2 -3 4 2 -1 1 -2 1 0 1 2 -2 -1 -1 -2 1 0 -1 1	3	3.0522	4	0.2413
536	0 2 -2 -2 2 0 -3 -2 -3 1 -1 0 0 -1 1 2 1 2 0 0 5	6	6.0637	7	0.228
537	0 2 -2 0 2 -2 0 -2 -2 1 -1 1 -1 -1 1 0 1 1 0 0 2	3	3.2361	4	0.25
538	0 2 -2 0 5 5 -2 -2 2 1 0 0 -1 -2 -3 1 -1 1 0 -5 0	1	1.2107	3	0.2346
539	0 2 2 -1 1 1 3 3 0 -2 0 -1 2 -3 0 -1 0 -1 -2 0 -3	0	0.0334	2	0.2183
540	0 2 2 1 -1 1 1 1 -1 1 -1 -1 -1 0 0 0 0 0 -2 -1 -1	0	0.1278	1	0.1768
541	0 2 2 1 -1 1 1 1 -1 1 0 -1 1 -1 0 -2 -1 0 -2 -1 0	1	1	2	0
542	0 2 2 1 1 -1 2 -2 1 1 0 -1 -2 -1 0 0 0 0 -2 0 -1	1	1	2	0

543	0 2 2 1 1 -1 3 -3 0 1 0 -1 -2 -1 0 -1 1 1 -2 0 0	2	2.0035	3	0.1258
544	0 2 2 1 3 -3 1 -1 1 0 0 0 -2 -2 1 1 -1 -1 -2 0 -1	0	0.365	2	0.2117
545	0 2 2 5 6 -7 5 -4 1 1 0 2 -1 -2 3 -4 0 -3 -2 -4 -1	3	3	5	0
546	0 3 -3 -3 3 0 -3 -3 -3 2 0 1 1 0 2 2 0 2 0 0 6	10	10.1105	12	0.1165
547	0 3 3 -1 1 1 4 5 -1 -2 0 -1 2 -4 0 -2 -1 -1 -3 0 -4	0	0	2	0
548	0 3 3 1 -2 1 1 1 -1 1 -1 -1 0 0 0 -1 0 -1 -3 0 -1	1	1	2	0
549	0 3 3 1 1 1 3 3 0 -2 0 -1 1 -3 1 -1 0 -2 -3 -2 -3	0	0	2	0
550	0 3 3 1 1 1 3 4 -1 -2 0 -1 1 -3 1 -1 -1 -1 -3 -2 -3	0	0	2	0
551	0 3 3 2 2 -2 3 -3 0 1 -1 -1 -2 -1 0 -1 1 1 -3 -1 0	2	2.0178	3	0.25
552	0 3 3 2 3 -3 2 -3 3 0 -1 -2 -2 -2 2 0 1 -1 -3 0 -2	1	1.1565	2	0.1695
553	0 3 3 2 4 -2 1 -1 -1 -1 -1 0 -2 -1 -1 1 -2 1 -3 -2 0	0	0.067	2	0.1508
554	0 3 3 3 -2 2 3 1 -3 1 -1 -1 -2 0 -1 -1 0 1 -3 -2 -1	1	1.0164	2	0.2309
555	0 3 3 3 -1 3 1 2 -2 1 -1 -2 -1 -1 -1 -2 -1 1 -3 -3 0	0	0	1	0
556	0 3 3 3 6 -3 2 -2 -2 -1 -1 1 -3 -2 -1 1 -2 1 -3 -3 0	1	1	3	0
557	0 3 3 5 4 -7 5 -2 3 1 -1 1 -1 -2 3 -4 0 -3 -3 -2 -3	2	2	4	0
558	0 3 3 5 5 -7 5 -3 2 1 -1 1 -1 -2 3 -4 0 -3 -3 -3 -2	2	2	4	0
559	0 4 4 1 2 -2 2 -2 1 1 -2 -2 -1 0 0 -1 0 1 -4 -1 -1	1	1	2	0
560	0 4 4 2 -4 4 1 0 -1 -1 -1 -2 -2 1 -2 1 1 -1 -4 0 0	1	1.1946	3	0.1836
561	0 4 4 2 -2 2 2 2 -2 1 -2 -2 -1 0 0 -1 0 0 -4 -2 -2	0	0	1	0
562	0 4 4 2 -2 2 2 4 -4 0 -1 -1 1 -1 -1 -3 -3 1 -4 -2 0	0	0.0393	2	0.124
563	0 5 5 -1 -1 1 -1 1 -2 -1 -2 -2 1 -2 1 1 1 -1 -5 0 1	1	1.0109	3	0.1264
564	0 5 5 1 -1 1 1 0 -1 1 -2 -2 -1 -2 1 -1 2 -2 -5 -1 0	0	0.0168	2	0.1472
565	0 5 5 3 -3 3 3 4 -4 1 -1 -2 1 -1 -1 -4 -3 1 -5 -3 0	1	1	3	0
566	0 6 6 1 1 -1 2 -1 -1 1 -2 -2 -1 1 -3 -1 -3 3 -6 -1 0	1	1	3	0
567	0 6 6 1 3 -3 2 -2 2 1 -3 -3 -1 0 0 -1 -1 1 -6 -1 -2	0	0	2	0
568	0 6 6 2 -2 2 2 2 -4 1 -2 -2 -1 -2 0 -1 0 0 -6 -2 0	1	1	3	0
569	0 7 7 1 3 -3 2 -2 2 1 -3 -3 -1 0 -1 -1 -2 1 -7 -1 -2	0	0	2	0
570	0 8 8 2 -2 2 1 3 -4 1 -3 -3 -1 -3 1 -1 0 -1 -8 -2 0	0	0	3	0
571	1 -1 -2 -1 2 1 -2 1 1 1 2 1 -1 -1 -2 1 -2 1 1 -1 0	3	3	4	0
572	1 -1 -2 -1 3 -3 -1 -3 -3 1 1 1 -1 -1 2 -1 1 1 1 0 3	5	5	6	0
573	1 -1 -1 -2 2 -1 -1 -2 -1 0 1 2 1 0 -1 0 0 0 1 0 2	4	4	5	0
574	1 -1 1 1 4 -4 3 -1 2 -2 2 2 1 -1 1 -2 -2 -3 0 -1 -2	2	2	4	0
575	1 -1 1 1 5 -5 3 -2 1 -2 2 2 1 -1 2 -2 -2 -3 0 -1 -1	3	3	5	0
576	1 -1 1 1 8 -8 3 -4 -1 -3 3 3 2 -2 4 -3 -2 -3 0 -1 1	6	6	9	0
577	1 -1 1 1 8 -8 3 -3 0 -3 3 3 2 -2 4 -3 -3 -4 0 -1 0	5	5	8	0
578	1 -1 1 2 0 2 1 0 -1 -3 2 0 0 -2 -1 2 2 0 0 -2 0	3	3	5	0
579	1 -1 1 3 8 -6 5 -3 2 -3 3 3 0 -2 2 -3 -3 -4 0 -3 -2	3	3	6	0
580	1 0 -1 -2 1 -1 2 1 1 1 1 0 0 1 1 -1 -2 0 0 1 -2	3	3	4	0
581	1 0 -1 -2 2 -1 -1 -2 -2 0 0 2 1 0 -1 0 0 0 0 0 2	3	3	4	0
582	1 0 -1 -2 2 -1 -1 -1 -1 0 0 2 1 0 -1 0 -1 -1 0 0 1	2	2	3	0
583	1 1 -2 -3 1 -3 -1 -3 -3 1 -1 1 1 1 1 -1 1 2 0 2 3	6	6	7	0
584	1 1 -2 -2 1 1 -3 -1 -3 0 0 0 0 0 0 2 0 2 0 0 4	5	5	6	0
585	1 1 -2 -2 2 -2 -3 -4 -2 2 -1 1 -1 1 1 0 1 1 0 0 4	5	5.0263	7	0.2163
586	1 1 -1 -6 2 -6 -1 -4 -3 3 -1 -1 2 1 2 -2 1 4 -1 4 4	9	9	11	0
587	1 1 -1 -5 2 -5 -2 -4 -2 3 -1 -1 1 1 2 -1 1 3 -1 3 4	8	8	10	0
588	1 1 -1 -5 2 -4 -3 -4 -1 3 -1 -1 1 1 2 0 1 2 -1 3 4	8	8	10	0
589	1 1 -1 -5 2 -3 -5 1 5 3 1 1 1 -2 -1 2 -2 -1 -1 3 0	6	6	8	0
590	1 1 -1 -5 2 -3 4 4 1 3 -1 -1 1 1 2 -3 -3 1 -1 3 -5	4	4	6	0
591	1 1 -1 -5 3 -4 -2 2 4 2 1 0 2 -1 1 0 -2 -1 -1 2 -2	4	4.2859	6	0.1825
592	1 1 -1 -5 4 -4 -1 -4 -3 2 0 -1 2 -1 1 -1 1 3 -1 1 4	6	6.2804	8	0.1734

593	1 1 -1 -5 5 -1 0 -2 -2 2 1 1 2 -2 -1 -2 -1 2 -1 0 2	4	4.0558	6	0.1579
594	1 1 -1 -5 6 -2 0 -2 -2 3 2 1 1 -2 1 -1 -2 1 -1 0 2	6	6	8	0
595	1 1 -1 -4 1 -4 -3 -4 -1 3 -1 -1 0 2 2 0 1 2 -1 3 4	8	8	10	0
596	1 1 -1 -4 1 -3 -4 1 4 2 1 1 1 -1 0 2 -2 -1 -1 3 0	5	5.1471	7	0.1509
597	1 1 -1 -4 2 -3 -2 -3 -1 2 -1 -1 1 0 1 0 1 2 -1 2 3	5	5.253	7	0.2063
598	1 1 -1 -4 2 -2 -3 -3 -1 2 -1 -1 1 0 1 1 1 2 -1 2 4	6	6.2398	8	0.2005
599	1 1 -1 -4 4 -4 -1 -4 -4 2 0 -1 1 -1 1 -1 1 3 -1 0 4	5	5.1721	7	0.1879
600	1 1 -1 -3 1 -3 -1 1 2 1 0 0 1 0 1 0 -1 0 -1 2 -1	3	3.0038	4	0.0626
601	1 1 -1 -3 1 -3 -1 2 2 1 1 0 1 1 1 0 -2 0 -1 2 -2	4	4	5	0
602	1 1 -1 -3 2 -3 -1 2 3 1 1 0 1 0 1 0 -2 -1 -1 1 -2	3	3	4	0
603	1 1 -1 -3 2 -2 -1 -2 -1 1 0 0 1 0 1 0 0 1 -1 1 2	4	4.0391	5	0.119
604	1 1 -1 -2 1 -2 -1 -2 -1 1 0 0 0 1 1 0 0 1 -1 1 2	4	4	5	0
605	1 1 -1 -2 5 3 -2 -2 2 2 1 0 -1 -2 -3 1 -1 1 -1 -3 0	2	2	4	0
606	1 1 2 1 1 2 4 4 -2 -2 1 -1 1 -2 -1 -2 -2 1 -2 -2 -3	0	0.0355	1	0.25
607	1 1 2 1 3 -3 2 -2 0 -1 0 0 0 -1 1 -1 0 -1 -2 -1 0	1	1	2	0
608	1 1 2 4 4 -5 3 -3 0 0 0 1 -1 -1 2 -3 0 -2 -2 -3 0	1	1.2736	3	0.1773
609	1 2 -2 -5 5 -5 -2 -5 -5 3 -1 -1 1 -1 1 -1 1 4 -1 0 5	7	7	9	0
610	1 2 -2 -3 5 2 -2 -2 2 0 0 -1 3 -2 1 -1 -1 -1 -1 -2 0	2	2.093	4	0.2118
611	1 2 -2 -2 2 -2 -2 -4 -2 2 -1 1 -1 1 1 0 1 1 -1 0 4	5	5.0279	7	0.2116
612	1 2 -2 -1 3 -3 -2 -3 -4 1 -1 0 -1 -1 2 1 1 2 -1 0 4	5	5	6	0
613	1 2 -2 -1 3 -3 0 -3 -3 1 -1 1 -1 -1 1 -1 1 2 -1 0 3	4	4	5	0
614	1 2 -1 -5 6 2 -2 -2 2 3 0 1 1 -2 -1 -1 -2 -1 -1 -2 0	3	3	5	0
615	1 2 2 1 -4 4 2 0 -2 -1 1 0 0 1 -1 -1 -1 -1 -3 -1 0	1	1.152	3	0.196
616	1 2 2 2 1 -2 3 -3 1 0 0 -2 -2 -1 2 -1 1 1 -3 0 -1	2	2	3	0
617	1 2 2 2 1 -2 3 -3 1 0 0 -1 -2 -1 1 -1 1 0 -3 0 -1	1	1	2	0
618	1 2 2 3 -2 1 3 2 -3 0 0 0 -2 0 -1 -1 0 1 -3 -1 -2	1	1	2	0
619	1 2 2 3 -2 3 3 1 -3 -1 0 -1 -1 0 -1 -2 -2 1 -2 -3 0	0	0	1	0
620	1 2 2 4 -4 4 4 1 -4 0 1 0 -1 1 -1 -3 -2 1 -3 -4 0	1	1.2159	3	0.1933
621	1 2 2 4 3 -5 4 -2 2 0 0 1 -1 -1 2 -3 0 -2 -3 -2 -2	1	1.3659	3	0.2012
622	1 2 2 4 4 -5 4 -3 1 0 0 1 -1 -1 2 -3 0 -2 -3 -3 -1	1	1.2955	3	0.187
623	1 2 3 1 6 -6 2 -2 -1 -1 0 1 0 -2 2 -1 -2 -1 -3 -1 0	2	2	4	0
624	1 2 3 2 5 -4 2 -2 0 -1 0 0 -1 -2 1 -1 -1 -1 -3 -2 0	0	0.3225	2	0.1917
625	1 2 3 2 6 -5 2 -2 -1 -1 0 1 -1 -2 1 -1 -2 -1 -3 -2 0	1	1	3	0
626	1 2 3 5 4 -7 5 -2 3 0 0 1 -1 -2 3 -4 0 -3 -3 -2 -3	2	2	4	0
627	1 2 3 5 6 -7 5 -4 1 0 0 1 -1 -2 3 -4 0 -3 -3 -4 -1	2	2	4	0
628	1 3 -3 -1 3 -3 -1 -4 -3 1 -2 1 -1 -1 1 0 2 2 -1 0 4	5	5	6	0
629	1 3 3 2 -5 6 2 0 -2 -1 1 0 -1 1 -2 -1 -1 -2 -4 -2 0	1	1	3	0
630	1 3 3 2 2 -3 3 -3 2 0 -1 -2 -2 -1 2 -1 1 0 -4 0 -2	1	1	2	0
631	1 3 3 3 1 -3 3 -3 2 0 -1 -1 -1 0 1 -2 1 -1 -4 -1 -1	1	1.0178	2	0.25
632	1 4 3 2 -2 2 2 3 -3 0 -1 -1 1 -1 -1 -3 -2 1 -4 -2 0	0	0.1265	2	0.2018
633	1 6 5 2 -2 2 2 5 -5 -1 -2 -1 1 -2 -1 -3 -3 1 -6 -2 0	0	0	2	0
634	2 1 -3 -2 2 -2 -3 -4 -3 1 -1 2 -1 1 1 0 1 1 0 0 4	5	5.1446	7	0.208
635	2 1 -3 -2 3 -3 -4 -5 -3 2 -1 2 -2 1 2 0 1 1 0 0 5	7	7	9	0
636	2 1 -1 -2 6 -5 -2 -2 -2 -1 1 4 1 -2 1 1 -2 -1 -1 0 2	5	5	7	0
637	2 2 -2 -6 2 -4 -4 2 4 2 1 1 2 -1 1 2 -3 -1 -2 4 0	7	7	9	0
638	2 2 -2 -6 4 -5 -1 -4 -3 2 -1 -1 2 -1 1 -1 1 4 -2 2 4	7	7.0094	9	0.076
639	2 2 -2 -6 4 -4 -2 -4 -2 2 -1 -1 2 -1 1 0 1 3 -2 2 4	7	7.0049	9	0.0702
640	2 2 -2 -6 5 -6 -1 5 6 2 1 0 2 -1 2 -1 -4 -2 -2 1 -5	4	4	6	0
641	2 2 -2 -6 6 -4 -2 -6 -4 2 0 -1 2 -2 1 0 2 3 -2 0 6	8	8	10	0
642	2 2 -2 -5 4 3 -5 -2 -5 1 0 -2 1 -2 -2 4 0 4 -2 0 7	8	8	10	0

643	2 2 -2 -5 5 -2 -1 3 4 2 1 2 1 -1 0 -1 -4 -2 -2 0 -3	3	3	5	0
644	2 2 -2 -4 4 -4 0 -4 -4 1 0 0 1 -1 1 -1 1 3 -2 0 4	5	5.2977	7	0.1778
645	2 2 -2 -4 6 2 -2 -2 2 2 1 1 0 -3 -3 0 -1 1 -2 -2 0	2	2.3913	5	0.25
646	2 2 -2 -2 3 -3 -1 2 2 1 1 2 -1 -1 1 1 -3 -2 -2 0 -1	2	2.0377	4	0.1528
647	2 2 4 4 6 -6 4 -4 0 -1 0 0 -1 -2 2 -3 0 -2 -4 -4 0	1	1	3	0
648	2 3 -3 -2 3 -3 -2 -5 -3 2 -1 2 -2 1 2 0 1 1 -2 0 5	7	7	9	0
649	2 3 3 2 -5 5 4 1 -4 -2 1 0 0 1 -1 -2 -2 0 -5 -2 0	2	2	4	0
650	2 3 3 6 5 -6 6 -5 1 0 0 1 -2 -1 2 -4 1 -2 -5 -5 -1	2	2	4	0
651	2 3 4 5 -5 5 5 2 -5 -1 1 -1 -1 1 -1 -4 -3 1 -5 -5 0	1	1	3	0
652	2 4 4 6 2 -4 6 -6 4 0 0 -1 -4 -2 1 -2 2 -1 -6 -2 -4	1	1	3	0
653	2 4 4 6 4 -4 6 -6 2 0 0 -1 -4 -2 1 -2 2 -1 -6 -4 -2	1	1	3	0
654	2 4 5 3 7 -5 3 -3 0 -2 -1 -1 -2 -3 1 -1 -1 -1 -5 -3 0	0	0	2	0
655	2 5 5 6 3 -5 6 -6 3 0 -1 -2 -4 -2 2 -2 2 -1 -7 -2 -3	1	1.0318	3	0.2291
656	2 5 7 6 5 -7 4 -5 3 -2 -1 -4 -4 -4 4 0 1 -1 -7 0 -2	2	2	4	0
657	2 6 6 4 -6 10 4 0 -4 -3 -1 -2 -3 1 -4 -1 -1 -2 -6 -4 0	0	0	3	0
658	2 6 6 6 4 -6 6 -6 4 0 -2 -3 -4 -2 3 -2 2 -1 -8 -2 -4	1	1.0391	3	0.2249
659	2 7 7 6 5 -7 6 -7 5 0 -3 -4 -4 -2 4 -2 3 -1 -9 -2 -4	2	2.0527	4	0.2253
660	2 7 7 9 4 -6 9 -9 5 1 -1 -3 -6 -3 2 -3 3 -2 -9 -4 -5	1	1.1066	4	0.2276
661	3 1 -4 -3 3 -3 -3 -5 -5 1 -1 3 -1 1 1 -1 1 2 0 0 5	7	7	9	0
662	3 2 5 3 6 -6 4 -4 -1 -2 0 -1 -1 -2 2 -2 0 -1 -5 -3 0	1	1.0502	3	0.234
663	3 3 -3 -7 8 -4 -2 4 6 3 2 3 1 -2 1 -1 -6 -3 -3 0 -4	5	5	8	0
664	3 3 -3 -4 8 4 -3 -3 3 2 1 1 -1 -4 -5 1 -1 2 -3 -4 0	2	2.6847	6	0.25
665	3 4 -4 -5 5 -5 0 -5 -5 1 -1 1 1 -1 1 -1 1 4 -3 0 5	7	7.0992	9	0.1211
666	4 4 8 6 10 -8 6 -6 0 -3 0 -2 -3 -4 2 -3 0 -2 -8 -6 0	0	0.1712	3	0.21
667	4 6 5 9 7 -9 9 -7 2 -1 -1 1 -3 -1 3 -6 1 -3 -9 -7 -2	2	2	5	0
668	0 0 0 0 0 0 0 0 0 0 -1 -1 -1 0 1 1 1 0 0 0 0	0	0	2	0
